# Programming Next‐Generation Synthetic Biosensors by Genetic Circuit Design

**DOI:** 10.1002/advs.202524172

**Published:** 2026-02-08

**Authors:** Yuanli Gao, Cheng Huang, Jiaxuan Deng, Lei Wang, Baojun Wang

**Affiliations:** ^1^ Key Laboratory of Biomass Chemical Engineering of Ministry of Education, College of Chemical and Biological Engineering Zhejiang University Hangzhou China; ^2^ ZJU‐Hangzhou Global Scientific and Technological Innovation Center Zhejiang University Hangzhou China; ^3^ Center of Synthetic Biology and Integrated Bioengineering & School of Engineering Westlake University Hangzhou China

**Keywords:** synthetic biosensor, genetic circuit design, cell‐free biosensor, whole‐cell biosensor, synthetic biology

## Abstract

Synthetic biology employs engineering principles to construct genetic circuits with customized functionality, empowering unprecedented control over biological systems. By harnessing this capability to precisely manipulate biological systems, synthetic biosensors are being developed as promising biosensing platforms for on‐site, sustainable, affordable, and easy‐to‐use detection across diverse scenarios, such as environmental monitoring, disease diagnosis, food safety control, and bioproduction optimization. However, the field deployment and real‐world application of synthetic biosensors face considerable challenges in biosensing sensitivity, specificity, speed, stability, and biosafety. This review summarizes recent advancements of genetic circuit‐enabled synthetic biosensors, focusing on their sensory mechanisms, designs, and applications. Moreover, the design principles, enabling tools, and engineering strategies for creating a high‐performing synthetic biosensor are analyzed. In particular, methods for tuning various characteristics of the dose‐response curve, including detection limit, detection threshold, operating range, dynamic range, and leakiness, are thoroughly examined. Finally, this review discusses the functional extension of biosensors by customizing signal‐processing and output modules, and outlines future directions to expedite the transition of synthetic biosensors from laboratory settings to field applications. Genetic circuit‐enabled synthetic biosensors, in collaboration with materials science, electronic engineering, and artificial intelligence, will tremendously expand the application space of synthetic biology.

## Graphical abstract

Synthetic biology enables genetic circuit‐based biosensing to detect diverse targets, process signals, and transduce them into readable outputs or intracellular regulatory activities. However, field deployment and real‐world application of such synthetic biosensors face considerable challenges in sensitivity, specificity, speed, stability, and biosafety. This review summarizes the sensory mechanisms, design principles, functional extensions, and future directions of genetic circuit‐enabled synthetic biosensors.

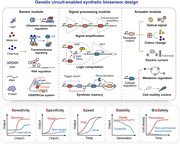

## 1 Introduction

Synthetic biology employs engineering principles to de novo construct biological systems or modify natural biological systems, creating sophisticated synthetic circuits with customized functions from modular genetic parts [1, 2]. Leveraging the capability of synthetic biology to precisely manipulate biological systems, synthetic biosensors are developed by rewiring or modifying natural biosensing pathways, or by rationally designing biorecognition elements [3, 4], as promising biosensing platforms to achieve sustainable, affordable, easy‐to‐use, and on‐site detection in diverse settings, such as environmental monitoring [5, 6], disease diagnosis [7, 8], food safety control [9, 10], and bioproduction optimization [11, 12].

**FIGURE 1:**
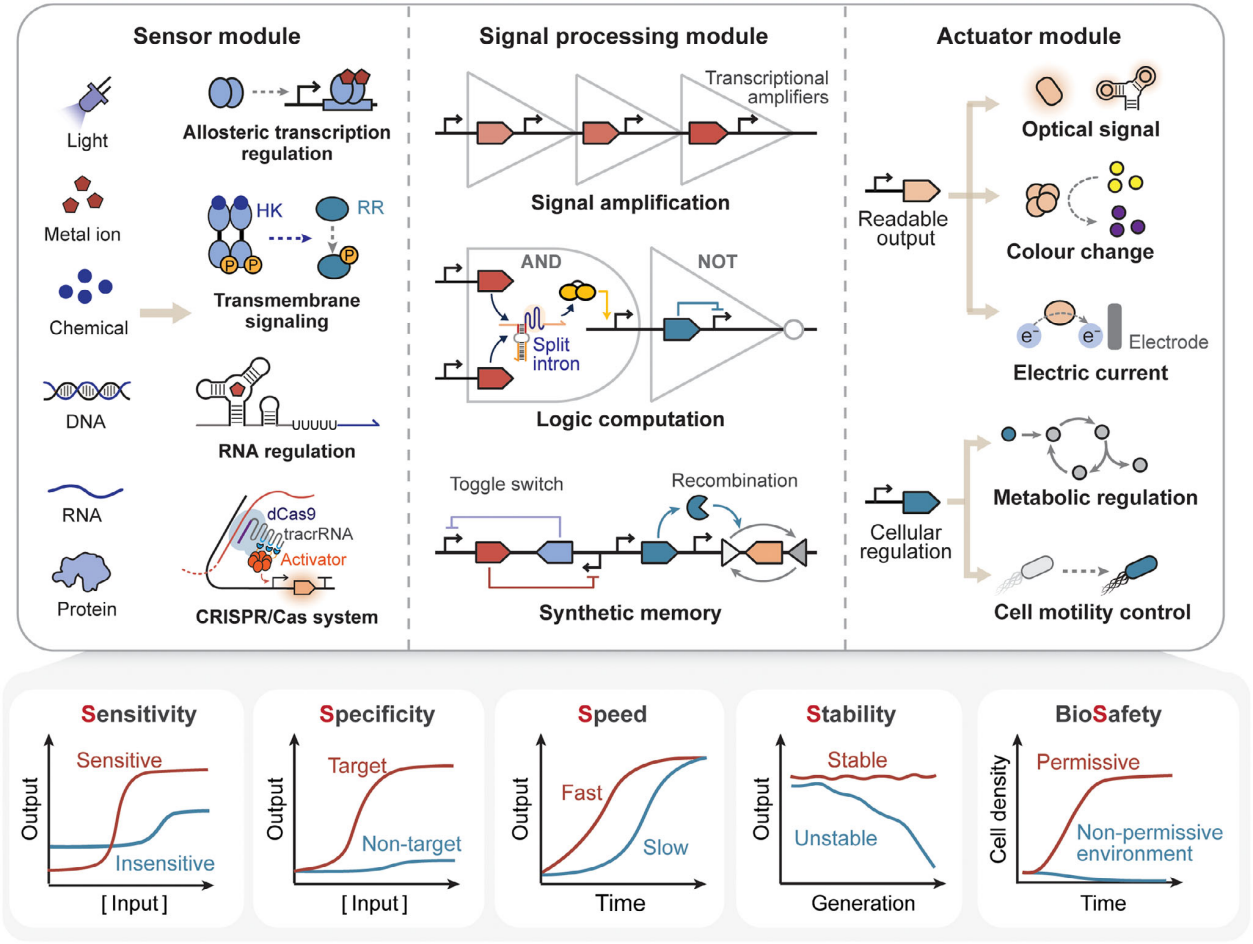
The anatomy (upper panel) and performance metrics (lower panel) of synthetic biosensors. Synthetic biosensors share a typical architecture comprising (1) the sensor module detects diverse input signals via various sensory modalities including allosteric transcription factors, transmembrane signaling pathways, riboswitches, and CRISPR/Cas systems; (2) the signal processor module reshapes the dose‐response and spatiotemporal properties of input signals, integrates multiple inputs by logic computation, records transient signals via synthetic memory devices; (3) the actuator module converts biosensors' responses into readable outputs or into regulatory activities for manipulating cellular processes. Biosensing performances can be evaluated across five dimensions: sensitivity, specificity, speed, stability, and biosafety.

Synthetic biosensors, including cell‐based and cell‐free biosensors, share a typical architecture comprising a sensor module, signal processing module, and actuator module (Figure 1, upper panel) [13]. The sensor module detects diverse input signals (e.g., light, metal ions, chemicals, nucleic acids, and proteins) via various sensory modalities, such as allosteric transcription factors, transmembrane signaling pathways, riboswitches, and CRISPR/Cas systems [14, 15, 16, 17]. The signal processor module reshapes the dose‐response and spatiotemporal properties of input signals, integrates multiple inputs by logic computation, records transient signals via synthetic memory devices, and transduces them to the actuator module [5, 18, 19]. The actuator module translates biosensors' responses into readable outputs (e.g., fluorescence, color change, and electric current) or into regulatory activities for manipulating cellular processes (e.g., protein secretion, biosynthesis, and chemotaxis) [11, 20, 21]. Compared with traditional instrumental analytical devices, synthetic biosensors are cost‐effective, easy to manufacture, and environmentally sustainable [22]. Notably, cell‐free biosensors can be embedded in paper or textile substrates or for portable distribution, freeze‐dried for long‐term storage, and rehydrated for target detection at the point of need, prolonging the shelf‐life and enabling on‐site detection of pathogenic nucleic acids and water contaminants [6, 7, 23, 24]. Freeze‐dried cell‐free components have been reported to retain transcription and translation activity after storage in tubes at room temperature for one year, although showing a nearly 80% decrease in output signals [25], and can be stored for even longer times at lower temperatures (e.g., 4°C) [26].

Despite the tremendous promise, synthetic biosensor development still faces considerable challenges towards the mission of field deployment and real‐world application. From an engineering perspective, biosensor performance can typically be assessed through dose‐response curve properties that reflect the input‐output relationship. In this framework, the challenges can be evaluated across five dimensions: sensitivity, specificity, speed, stability, and biosafety (Figure 1, lower panel). An ideal synthetic biosensor should be capable of detecting low concentrations of target ligands [27] and rapidly generating clear outputs in response to small changes in ligand concentration [28], which is particularly important for applications such as disease diagnosis and environmental monitoring. In complex environmental settings, biosensors need to accurately distinguish the target signal from non‐target signals [14] while maintaining stable performance and genetic integrity [29]. Lastly, the engineered cells should not survive when escaping into non‐permissive environments, crucial for ensuring the biosafety of synthetic biosensors [30].

This review focuses on current advancements in the development of synthetic biosensors enabled by genetic circuits, highlighting their sensory mechanisms, designs, and applications. This review further analyzes the design principles, enabling tools, and engineering strategies to design a high‐performing synthetic biosensor in terms of sensitivity, specificity, speed, stability, and biosafety. In particular, we thoroughly examine strategies for tuning various characteristics of the dose‐response curve, including detection limit, detection threshold, operating range, dynamic range, and leakiness. Finally, we discuss the functional extension of biosensors by customizing signal processing and output modules to expedite the transition of synthetic biosensors from laboratory settings to field applications.

## 2 Programming Synthetic Biosensors by Genetic Circuit Design

This section reviews the state of the art in synthetic biosensor development and provides an in‐depth analysis of the design strategies and applications of genetic circuit‐enabled biosensors (Table 1). Based on their regulatory mechanisms, the synthetic biosensors discussed here are classified into four categories: allosteric transcription regulation, transmembrane signal transduction, RNA regulation, and CRISPR/Cas systems.

**TABLE 1:** Typical genetic circuit‐enabled synthetic biosensors and applications.

Application		Target signal	Genetic component^a^	Limit of detection^b^	Operating range	Ref
Environmental monitoring	Heavy metal detection	As(III), Hg(II)	ArsR allosteric transcription factor (aTF) coupled with a transcriptional amplifier	0.1 ppb As(III), 0.01 ppb Hg(II)	0.1–5 ppb As(III), 0.01–10 ppb Hg(II)	[5]
		Cd(II)	CadR aTF	0.39 µg/L	0–60 µg/L	[31]
		As(III)	Reprogrammed tracrRNA that hijack endogenous mRNA as crRNA to activate gene expression in CRISPRa system	NA^c^	0–32 µM	[16]
		U(VI)	UzcRS‐UrpRS two‐component systems (TCS) coupled with the genetic AND gate and UzcY signal amplifier	1 µM	1–5.2 µM	[32]
		Hg(II)	MerR aTF coupled with toehold switch	5 nM	5–7.5 nM	[33]
		Au(III)	HspR aTF coupled with recombinase	5 µM	5–100 µM	[34]
		Cu(II)	CusRS TCS	12 µM	0.012–2 mM	[35]
		Zn(II)	ZraRS TCS and ZntR aTF integrated by a genetic AND gate	100 µM	0.1–1.2 mM	[35]
	Explosive residue detection	2,4‐dinitrotoluene (2,4‐DNT)	*yqjF* promoter	4.8 mg/L	4.8–25 mg/L	[36]
		2,4,6‐trinitrotoluene (TNT)	TNT riboswitch coupled with recombinase	25 µM	25–35 µM	[37]
	Pesticide detection	2‐phenylphenol (2‐PP)	HbpR aTF coupled with a transcription amplifier	1 µM	1–50 µM	[38]
	Organic contaminant detection	Monocyclic aromatic hydrocarbons	TodTS TCS	0.04 mg/L	0.04–1 mg/L	[39]
		Methanol	MxcQZ‐OmpR chimeric TCS	NA	0–0.05%	[40]
	Bacterial pathogen detection	N‐acyl‐homoserine lactone (AHL)	QscR aTF	0.01 µM	0.01–5 µM	[41]
Disease diagnosis and treatment	Body fluid monitoring	Progesterone	De novo designed transcription factor DLA	0.16 µg/L	0.16–60 µg/L	[42]
		Zinc in human serum	ZntR aTF and Zur aTF	NA	0–20 µM	[43]
		Cannabinoid	Human CB_2_ receptor integrated into yeast G‐protein coupled receptors (GPCR) signaling pathway	1 nM	1 nM–10 µM	[44]
		Uric acid	HucR aTF coupled with a trans‐splicing denoiser and a uric acid transporter UacT	1.56 µM	1.56–100 µM	[45]
		L‐lactic acid	LldR aTF coupled with a trans‐splicing denoiser and a lactate permease LldP	0.25 mM	0.25–40 mM	[45]
	Disease biomarker detection	Transforming growth factor‐β (TGF‐β)	Smad‐responsive promoter for gene expression in mammalian cells	0.024 ng/mL	0.024‐6.25 ng/mL	[46]
		Bile salts	Artificial transmembrane transcription factor CadC‐TcpP	28.3 µM	28.3–58.99 µM	[47]
		Heme	HrtR aTF coupled with a toggle switch	0.019 µM	0.019–0.1 µM	[48]
		Nitric oxide (NO)	NorR aTF coupled with recombinase	20 µM	20–100 µM	[49]
		Hydrogen peroxide (H_2_O_2_)	OxyR aTF coupled with recombinase	0.1 µM	0.1–20 µM	[49]
		Thiosulfate (S_2_O_3_^2−^)	ThsRS TCS coupled with recombinase	10 µM	10–200 µM	[49]
		Tetrathionate (S_4_O_6_^2−^)	TtrRS TCS coupled with recombinase	1 µM	1–100 µM	[49]
		RNA	CRISPR‐Cas13a/C2c2 system	NA	NA	[50]
		DNA	CRISPR‐Cas12a system	NA	NA	[51]
	Cell‐based therapeutics with smart control	Fatty acid	Artificial mammalian transcription factor LSR engineered by fusing PPARα ligand‐binding domain to TtgR DNA binding domain	5 µM	5–100 µM	[52]
		Cholera autoinducer 1 (CAI‐1)	*L. lactis* hybrid receptor fusing CqsS trans‐membrane ligand binding domain to the NisK signal transduction domain	*V. cholerae* with cell density of 10^8^ CFU/ml	NA	[20]
		Protocatechuic acid (PCA)	PcaV aTF	NA	0–1000µM	[53]
		Thiosulfate (S_2_O_3_^2−^)	ThsRS TCS coupled with base editor	0.016 mM	0.016–1 mM	[54]
		Aspirin	Artificial mammalian transcription factor Myr‐NPR1/NPR4‐VanR‐VP16	10 µM	10–250 µM	[55]
		Nitroglycerin	Artificial metabolic pathway converting NG to cGMP, which activates PKG1 to phosphorylate mammalian endogenous transcription factor CREB	23 µM DETA NONOate	23–150 µM DETA NONOate	[56]
	Modulation of the gut ecosystem	Rhamnose (Rha), chondroitin sulfate (ChS), arabinogalactan (AG), IPTG	RhaR aTF, LacI aTF, and putative hybrid TCSs BT3334 and BT0267 coupled with recombinase and CRISPRi in *B. thetaiotaomicron*	10 µM Rha, 0.0016% ChS 0.0005% AG; 0.5 µM IPTG	10–370 µM Rha, 0.0016–0.04% ChS, 0.0005–0.4% AG, 0.5–8 µM IPTG	[57]
Biomanufacturing	High‐throughput screening for desirable strains	L‐lysine	LysG aTF in *C. glutamicum*	40 mM	40–320 mM	[58]
		L‐cysteine	CcdR aTF in *E. coli*	0.2 mM	0.2–50 mM	[59]
		Malate	MalR aTF in *B. licheniformis*	5 g/L	5–15 g/L	[60]
	Dynamic regulation of metabolic pathways	Galacturonate	ExuR aTF in *E. coli*	NA	1–100 mg/L	[61]
		Glucose	Transcription factor Mlc that can be recruited to cell membrane during glucose uptake in *E. coli*	NA	NA	[11]
		L‐lysine	LysG aTF coupled with CRISPRi in *E. coli*	0.5 mM	0.5–8 mM	[62]
	Optimization of bioprocess parameters	L‐lactic acid, D‐lactic acid	LldR aTF and PdhR aTF in *Lactobacillus* species	NA	15–200 mM L‐lactic acid, 0–50 mM D‐lactic acid	[63]
	Directed evolution of enzymes	Lactulose	LacI‐L5 aTF with altered effector specificity	5 µM	5–500 µM	[64]
		Theophylline	Theophylline riboswitch	10 µM	10–1000 µM	[65]
		Alkaloids	RamR aTFs evolved to specifically detect five alkaloids (THP, PAP, ROTU, GLAU and NOS)	1 µM	1–100 µM	[14]
		4'‐O‐Methylnorbelladine (4NB)	RamR aTF evolved into a biosensor for 4NB	2.5 µM	2.5–100 µM	[66]
Spatiotemporal regulation of cell behavior	Regulation of cell division and movement	Light	Artificial aTF LexA‐RsLOV coupled with genetic AND, NAND, NIMPLY, and OR gates	0.01 mW/cm^2^	0.01–0.41 mW/cm^2^	[67]
	Regulation of bacterial swarm patterns	Cu(II)	Native *P. mirabilis* P*_copA_* promoter regulating *flgM* gene	10 mM	10–50 mM	[21]
Food safety	Food toxin detection	Putrescine	PuuR aTF in *E. coli* and cell‐free system	5.37 mM in *E. coli* 4.33 mM in cell‐free system	5.37‐1000 mM in *E. coli*, 4.33‐100 mM in cell‐free system	[68]
		Toxoflavin	ToxR aTF in *B. glumae*	50 nM	50–2000 nM	[9]
		Histamine	HinK aTF in *P. putida*	0.39 ppm	0.28–18 ppm	[10]
		Tetracycline	TetR aTF coupled with the amplifier circuit based on polymerase strand recycling in cell‐free system	25 nM	25−100 nM	[27]
	Allergen detection	Parvalbumin	CD63‐EGFP fusion protein expressed in response to fish allergen Parvalbumin on mast cell surfaces	1 ng/mL	1–100 ng/mL	[69]

^a^ Abbreviations: aTF, allosteric transcription factor; *B. glumae*, *Burkholderia glumae*; *B. licheniformis*, *Bacillus licheniformis*; *B. thetaiotaomicron*, *Bacteroides thetaiotaomicron*; *C. glutamicum*, *Corynebacterium glutamicum*; *L. lactis*, *Lactococcus lactis*; *P. mirabilis*, *Proteus mirabilis*; *P. putida*, *Pseudomonas putida*; TCS, two‐component system; *V. cholerae*, *Vibrio cholerae*.

^b^ Units: CFU, colony‐forming units; ppm, parts per million; ppb, parts per billion.

^c^ NA, not available. The data was not reported in the original literature.

### 2.1 Biosensors Based on Allosteric Transcription Regulation

#### 2.1.1 Allosteric Transcription Factors

**FIGURE 2:**
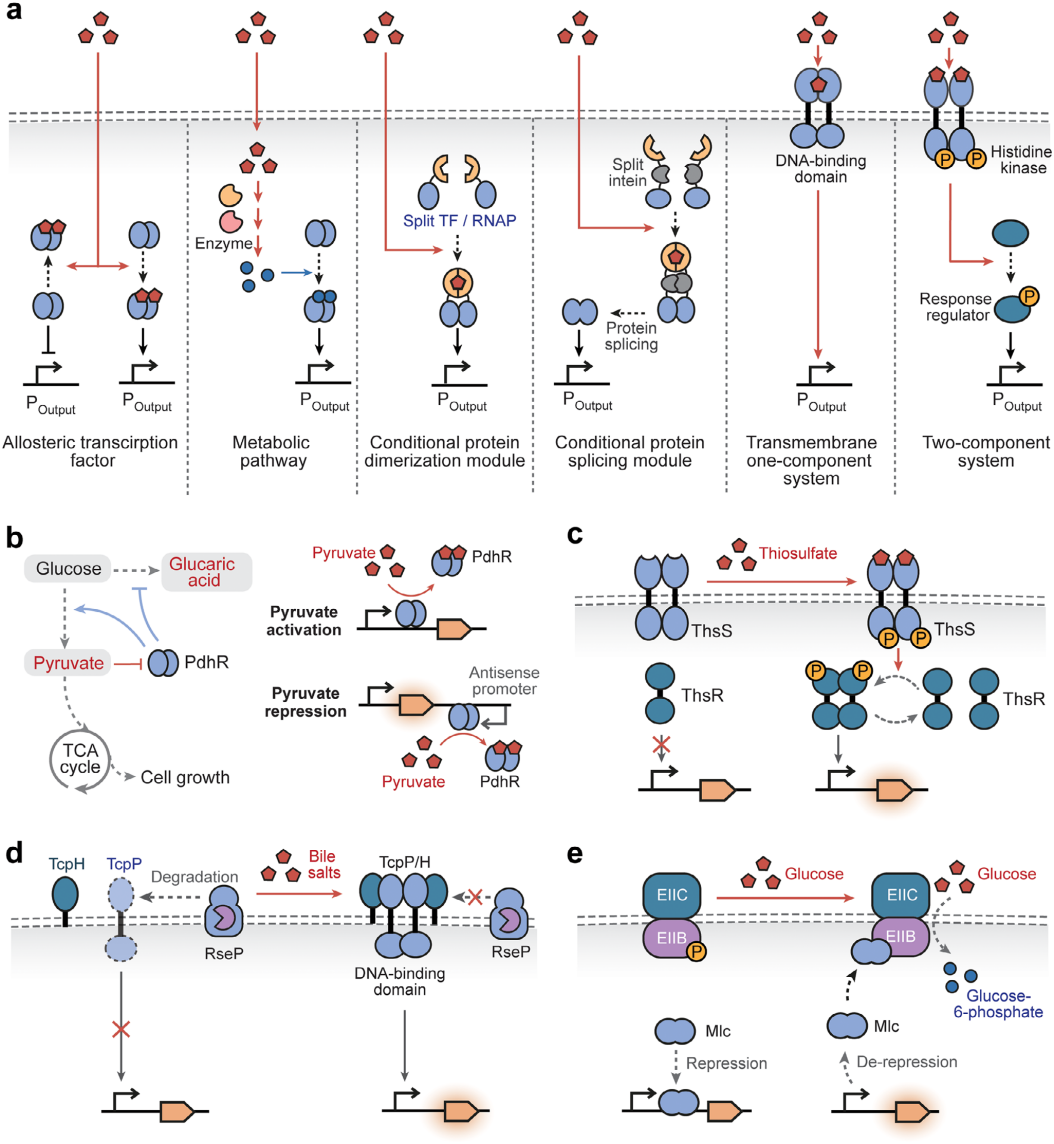
Synthetic biosensors based on transcription regulation. (a) Common types of the sensory mechanisms of synthetic biosensors based on transcription regulation, including allosteric transcription factors (aTFs), biosensing‐enabling metabolic pathways, conditional dimerization or splicing of split transcription regulators (e.g., transcription factors and RNA polymerases), transmembrane one‐component system, and two‐component systems (TCSs). TF, transcription factor. RNAP, RNA polymerase. (b) Pyruvate‐responsive biosensors for bifunctional regulation of metabolic pathways. [12] For pyruvate‐triggered gene activation, the transcription repressor PdhR disassociates from its cognate promoter upon pyruvate binding for transcription depression. For pyruvate‐triggered gene repression, PhdR regulates the antisense promoter and the binding of pyruvate activates antisense transcription for target gene inhibition. (c) Thiosulfate‐responsive TCS biosensor in which histidine kinase ThsS phosphorylates response regulator ThsR for gene activation. [49, 54] (d) Bile salt‐responsive biosensor based on the transmembrane one‐component system. In the presence of bile salts, the CadC‐TcpP receptor dimerizes and forms a stable complex with TcpH, protecting the receptor from RseP‐mediated proteolysis and activating expression of the output reporter. [47] (e) Phosphotransferase system‐based biosensor for monitoring glucose uptake rate. The transcription repressor Mlc inhibits its cognate promoter in the absence of glucose, and is recruited to the membrane‐bound unphosphorylated EIIB in the presence of glucose. [11]

Allosteric transcription factors (aTFs) are DNA‐binding proteins that regulate the transcription activities of target promoters in response to specific ligands. A typical aTF consists of two functional domains, a ligand‐binding domain and a DNA‐binding domain. The selective recognition of the target ligand by the ligand‐binding domain triggers a conformation change in the DNA‐binding domain, hence altering the affinity of the aTF for the target promoter to activate or inhibit downstream gene expression (Figure 2a).

##### 2.1.1.1 Regulatory Mechanisms of aTF‐Based Biosensors

In aTF‐based biosensors, the expression of output genes is modulated via three mechanisms: transcription activation, repression, or bifunctional regulation. Ligand‐triggered transcription activation of the target promoter is the predominant mechanism for developing aTF‐based biosensors, which can be achieved through transcription activators or repressors. Transcription activators (e.g., quorum‐sensing molecule 3OC_6_HSL‐responsive LuxR) [35] bind to their target promoters in the presence of target ligands and recruit RNA polymerase (RNAP) to evoke the transcription process. By contrast, transcription repressors (e.g., arsenic‐responsive ArsR) [70] bind to the promoter to obstruct RNAP access to the transcription initiation site, and disassociate from the target promoter upon ligand binding for transcriptional derepression.

Ligand‐triggered transcription repression of target gene expression has also been engineered by using antisense promoters or anti‐repressors. For instance, pyruvate‐responsive PdhR was employed to regulate the antisense promoter of a reporter gene (Figure 2b) [12]. In the absence of pyruvate, PdhR binds its target promoter to suppress antisense transcription, thereby activating target gene expression. In the presence of pyruvate, PdhR dissociates from the antisense promoter, releasing antisense transcription to inhibit target gene expression. This pyruvate‐inhibited switch, together with the pyruvate‐activated sensor, was used to dynamically reallocate metabolic flux between the glucaric acid synthesis pathway and the central carbon metabolism, enhancing the bioproduction yield of microbial factories. Alternatively, ligand‐triggered gene repression can be implemented using anti‐repressors (e.g., TrpR) [71] with increasing affinity for target DNA upon ligand binding [72]. More specifically, TrpR cannot bind to its target promoter for transcriptional suppression until the addition of L‐tryptophan (L‐Trp) [71]. The trpR‐based whole‐cell biosensor was leveraged to report the catalytic activities of tryptophan hydroxylase variants, which convert L‐Trp to 5‐hydroxytryptophan (5‐HTP), a precursor of the neurotransmitter serotonin.

Bifunctional aTFs form repressive complexes to inhibit the target promoter without the target ligand, but activate promoter transcription upon ligand binding. One noteworthy example is the lactate‐responsive LldR [45], employed to develop lactate biosensors to detect physiological biomarkers [45], optimize fermentation parameters [63], and monitor mammalian cell cultures during biopharmaceutical production [73]. In the absence of lactate, LldR dimers bind to two operator sites (O1 and O2) in the P*_lldP_* promoter to form a tetramer, leading to DNA looping that sequesters the promoter and suppresses transcription initiation. Once lactate binds to LldR, the tetramer complex is disrupted, and LldR dimers bound to O1 activate the transcription of the downstream gene [73].

##### 2.1.1.2 Strategies for Engineering aTF‐Based Biosensors

Strategies for developing aTF‐based biosensors include mining natural aTFs, rationally engineering artificial aTFs, and integrating biosensing‐enabling metabolic pathways. The sophisticated natural transcription regulatory networks have provided a wealth of aTFs with diverse ligand‐responsive capabilities for biosensor development. So far, more than 20 families of prokaryotic aTFs have been discovered, including the AraC, ArsR, LacI, LysR, TetR, and LuxR families, which are involved in various cellular processes such as metal homeostasis, sugar metabolism, antibiotic resistance, and quorum sensing [74]. Engineering aTF‐based biosensors for specific ligands has also been accelerated by systematic methodologies for natural aTF identification, including transcriptome analysis [75], promoter screening [76], genome mining [77], and implementation of aTF prediction tools (e.g., JASPAR [78], MotifMap [79], and Sensbio [80]), as well as databases (e.g., PRODORIC [81], RegulonDB [82], AnimalTFDB [83], and GroovDB [84]).

Rational engineering of artificial aTFs further expands the sensing and regulatory capabilities of biosensors beyond the naturally occurring aTFs. As a classic example, Scholz et al. demonstrated that a single amino acid substitution could lead to the functional reversal of TetR, converting its target ligand anhydrotetracycline (aTc) from an inducer to a co‐repressor [85]. Rational design of aTFs with varying specificities for non‐natural ligands can be facilitated by computational design methods that identify critical residues in the ligand‐binding pocket and in silico predict beneficial mutants with higher ligand‐binding affinities [86]. Computational protein design tools, such as Rosetta and Phoenix Match, have been leveraged to alter the binding specificity of aTFs (e.g., LacI, PobR, and QacR) towards non‐native ligands for biosensing in *E. coli*, yeast, and cell‐free systems [87, 88, 89]. The DNA recognition and allosteric properties of aTFs can be altered by domain swapping or fusion. The ligand‐ and DNA‐binding domains from different aTFs could be swapped to generate novel hybrid aTFs with rewired input‐output relationships, which have been validated in aTF families including LacI [72], LuxR [90], MerR [91], and TetR [92], and applied to execute multi‐input logic computation [93], rewire natural product regulation [90], and monitor environmental heavy metal contamination [91]. Furthermore, bacterial‐derived aTF could be fused to eukaryotic transactivation domains (e.g., VP16 or VP64) or additional ligand‐binding domains to create biosensors functional in eukaryotic systems. In a recent study, a mammalian biosensor for the sigma factor X‐inducing peptide (XIP) was engineered by fusing bacterial aTF ComR to a mammalian transactivation domain [94]. Likewise, the bacterial vanillic‐acid‐responsive VanR was appended to the *Arabidopsis thaliana*‐derived aspirin‐responsive NPR4 domain and to the VP16 transactivation domain for aspirin biosensing in mammalian cells [55]. However, it remains challenging to rationally design high‐performing aTFs, especially to achieve high‐affinity binding for non‐natural ligands without arduous experimental screening, overcome cellular toxicity or resource depletion caused by high aTF expression, and minimize cross‐talk with endogenous cellular pathways.

The chemical space detectable by biosensors can be expanded by integrating metabolic pathways that convert undetectable molecules into metabolites easier to detect using biosensors (Figure 2a). For instance, synthetic metabolic cascades could convert cocaine and hippuric acid into benzoic acid, which can be detected by BenR‐based whole‐cell [95] and cell‐free biosensors [96], enabling detection of drugs (cocaine) and metabolites (hippuric acid) in clinical urine samples. Likewise, clinically relevant metabolites (lactate, sarcosine, and choline) can be metabolized to hydrogen peroxide and reported by OxyR‐based biosensing circuits in cell‐free systems [97]. Moreover, several computational tools have been developed to automatically design biosensing‐enabling metabolic pathways, such as SensiPath [98] and BioSensor Galaxy workflow [97].

#### 2.1.2 Conditional Dimerization or Splicing of Split Transcription Regulators

Novel sensing functions of transcription regulators can be engineered by incorporating synthetic ligand‐sensing domains to regulate the dimerization or splicing of split proteins (Figure 2a). In recent years, various conditional dimerization domains responsive to light, temperature, and chemicals have been integrated with transcription regulators such as RNA polymerases, transcription factors, recombinases, and CRISPR/dCas9 systems [67, 99, 100, 101]. The most noticeable example is the conditional dimerization of split T7 RNA polymerase (RNAP), where a proximity‐dependent split T7 RNAP was obtained through continuous molecular evolution and appended with conditional dimerization domains to activate target gene transcription upon sensing light [102], rapamycin [102], and temperature change [99]. This scheme was further extended by target‐dependent RNAP (TdRNAP), which recognizes target molecules via the variable domains (V_H_ and V_L_) of the single antibody to trigger split T7 RNAP assembly [103]. It is noteworthy that TdRNAP can be modularly assembled with various antibodies against the FLAG peptide, EGFP, HCV IRES RNA, and fluorescein, capable of transducing peptides/proteins, RNAs, and small molecules into transcriptional signals in mammalian systems. Similarly, split T7 RNAP halves were fused to different affinity domains, namely nanobodies, monobodies, and DARPins (designed ankyrin repeat proteins), against the same protein target (e.g., mCherry, SARS‐CoV‐2 receptor binding domain, and transthyretin), which colocalize the T7 RNAP halves to trigger LacZ expression and produce colorimetric output in the cell‐free system [104].

Conditional intein‐mediated protein splicing modulates the activity of transcription regulators through peptide ligation in response to input signals. During protein splicing, split inteins excise themselves from precursor peptides and covalently ligate the flanking protein segments (exteins) [105]. Switchable intein splicing could be engineered by appending ligand‐binding domains (e.g., rapamycin‐responsive FKBP‐FRB dimerization domains) [106] to colocalize the split intein halves, leaving minimal scars after protein splicing to reduce the chance of compromising original protein function caused by additional domain insertion. Our group has developed a transposon‐based approach to identify optimal insertion sites for ligand‐binding domains within specific inteins [107]. A caffeine‐responsive biosensing circuit was created by grafting acVHH domains into M86 intein‐split transcription factor ECF20. In the presence of caffeine, the acVHH domains could homodimerize to trigger protein splicing, producing ECF20 to activate downstream gene expression.

### 2.2 Biosensors Based on Transmembrane Signal Transduction

Transmembrane signaling systems receive extracellular chemical or physical stimuli and transduce these input signals into intracellular responses, providing a wealth of sensing modalities that can be programmed into synthetic biosensors. In this section, we reviewed synthetic biosensors derived from classic transmembrane systems, the two‐component system, and recent processes in devising biosensors based on novel mechanisms, the transmembrane single‐component system and phosphotransferase system, with a focus on prokaryotic biosensors (Figure 2a). Notably, eukaryotic transmembrane signaling, such as G‐protein‐coupled receptor (GPCR) pathways [4], has also been intensively investigated and engineered into synthetic biosensors for therapeutic applications, as systematically summarized in recent reviews [108, 109].

#### 2.2.1 Two‐Component System

The two‐component system (TCS) is a ubiquitous family of transmembrane signaling systems discovered in bacteria, archaea, and non‐animal eukaryotes, constituting the most extensive category of multi‐step signal transduction pathways in nature [110]. The canonical TCS sensor consists of a transmembrane histidine kinase (HK) for signal sensing, and a cytoplasmic response regulator (RR) for transcriptional regulation (Figure 2a). Upon sensing an input signal, the periplasmic sensory domain of HK undergoes a conformational change, which is relayed to the transmitter domain for autophosphorylation. The phosphoryl group is then transferred to an aspartate residue within an N‐terminal receiver domain of RR. The phosphorylated RR exhibits activated DNA‐binding activity, typically via receiver‐domain‐mediated dimerization, to modulate the transcription of its cognate output promoter [111]. In the absence of input signals, HKs exhibit phosphatase activity, dephosphorylating and inactivating their cognate RRs, thereby suppressing TCS sensor outputs [112]. Therefore, TCS performance can be fine‐tuned by adjusting the relative balance of the HK's kinase and phosphatase activities. For instance, the detection thresholds of TCS biosensors were shown to increase with HK phosphatase activity [113].

TCSs can detect a wide array of inputs (e.g., hormones, pH, metals, temperature, and small‐molecule metabolites), which have been utilized for biosensor development applicable to cellular regulation, environmental monitoring, as well as disease diagnosis and treatment [110]. TCSs have exhibited remarkable capabilities for sensing light across a broad spectrum of wavelengths, including UV‐violet [114], blue, green, red [115], and near‐infrared light [116]. Light‐responsive TCS sensors were applied to exert spatiotemporal control over cellular gene expression [115], pattern cell adhesion onto various materials (e.g., textiles, ceramics, and plastics) [117], and dynamically regulate metabolic pathways [120]. For environmental monitoring, TCS sensors based on CusRS and ZraRS systems can detect copper and zinc for the assessment of heavy metal contamination [35]. Moreover, TCS sensors based on the UrpRS and UzcRS systems have been developed in *Caulobacter crescentus* for the specific recognition of uranium, a radionuclide, in groundwater samples [32]. For biomedical applications, ThsRS‐based TCS sensors were implemented to detect thiosulfate, a biomarker for inflammatory bowel disease (Figure 2c) [49, 54]. Thiosulfate at disease‐relevant concentrations could activate expression of the recombinase or base editor for genetic memory of input signals, or trigger the secretion of the therapeutic protein AvCystatin to ameliorate inflammation [49, 54]. Additional examples include the TtrRS‐based TCS biosensor for the detection of tetrathionate, another inflammation biomarker [8], and the TorRST‐based TCS biosensor for trimethylamine N‐oxide (TMAO), a biomarker for cardiovascular disorders [121].

Natural TCS systems often exhibit limited cross‐host portability and elevated basal leakage expression associated with their native promoters. Several strategies have been developed to tackle this challenge, including promoter engineering and domain swapping. Promoter engineering strategies reassemble RR‐binding sites with synthetic promoters tailored for specific hosts or featuring improved dynamic ranges [114]. For instance, the MalKR‐based TCS has been successfully transported into *Saccharomyces cerevisiae* by designing artificial hybrid promoters containing MalR‐binding sites within a yeast promoter for monitoring L‐malic acid bioproduction [122]. Furthermore, the DNA‐binding domains (DBDs) of TCS response regulators (RRs) could be modularly substituted with those with well‐defined characteristics and high‐performing cognate promoters [15]. Utilizing this method, Schmidl et al. swapped the DBDs of seven uncharacterized TCSs from *Shewanella oneidensis* with PsdR DBD to screen their ligand‐responsive activities in *E. coli* [15]. Nonetheless, TCS‐based biosensors are still hindered by several issues, including the reliance on intracellular ATP supply for signal transduction, potential crosstalk among different TCSs, and a lack of systematic methodologies for porting TCSs into eukaryotic systems.

#### 2.2.2 Transmembrane Single‐Component System

The transmembrane single‐component system is the simplest form of bacterial transmembrane signal transduction. The transmembrane single‐component system functions through ToxR‐family receptors, which consist of an N‐terminal cytoplasmic DNA‐binding domain, a transmembrane domain, and a C‐terminal periplasmic sensory domain [123]. During signal transduction, the input signal perceived by the sensory domain is transduced through the transmembrane domain to the DNA‐binding domain, activating the transcription of the output promoter (Figure 2a).

The ToxR family activator CadC, responsive to both pH and lysine, has been developed into lysine biosensors for dynamic regulation of cadaverine biosynthesis in *E. coli* [124]. In the absence of lysine, CadC binds to LysP, a lysine‐specific transporter, inhibiting its own DNA‐binding activity. In the presence of lysine in acidic environments, CadC dissociates from LysP and binds its cognate promoter to activate output gene expression. The pH‐sensing residues of CadC were then mutated to generate a pH‐independent lysine biosensor.

The ligand specificity of transmembrane single‐component systems can be altered by replacing the sensory domains. Chang et al. first replaced the CadC sensory domain with a caffeine‐induced dimerization domain to design a hybrid transmembrane receptor [123]. In the following study, they rewired the TcpP sensory domain to the CadC DNA‐binding domain, to engineer a CadC‐TcpP hybrid receptor for detecting bile salts in clinical serum samples (Figure 2d) [47]. In the presence of bile salts, the CadC‐TcpP receptor dimerizes and forms a stable complex with TcpH, protecting the receptor from RseP‐mediated proteolysis. The Cad‐TcpP dimer then binds the CadC‐cognate promoter and activates the output reporter expression.

#### 2.2.3 Phosphotransferase System‐Based Biosensors

The bacterial phosphotransferase system (PTS) has been discovered to transport and phosphorylate over 20 carbohydrates into bacterial cells and to transduce chemical inputs into intracellular signals, regulating various biological processes such as iron homeostasis, pathogen virulence, and stress response [125]. PTS comprises three functional components: phosphotransferase enzyme I (EI), histidine phosphate carrier protein HPr, and enzyme II (EII) complex, which consists of EIIA, EIIB, EIIC, and sometimes EIID domains or proteins.

Recently, the *E. coli* PTS has been rewired to develop a biosensor for monitoring glucose uptake rate [11]. The signal transduction from glucose signal to output gene expression is executed by Mlc, a transcription repressor that inhibits its cognate promoter in the absence of glucose, and is recruited to the membrane‐bound unphosphorylated EIIB in the presence of glucose (Figure 2e). This biosensor for glucose uptake rate has been leveraged to implement feedback‐loop control systems that dynamically regulate metabolic pathways for enhancing the biosynthesis of L‐tryptophan, riboflavin, and D‐lactic acid.

### 2.3 Biosensors Based on RNA Regulators

#### 2.3.1 Riboswitches

Riboswitches are cis‐acting RNA regulatory elements located within the untranslated regions (UTRs) of a target gene. The riboswitch is typically composed of an aptamer domain for ligand recognition and an expression platform to regulate downstream gene expression. The specific ligand binding onto the aptamer domain evokes a conformational change, transmitted to the regulatory elements (e.g., terminator, ribosome‐binding site, and ribozyme) in the expression platforms. Therefore, riboswitches can alter gene expression by various mechanisms, including transcription termination control, translation initiation regulation, RNA cleavage, RNA splicing, and RNA editing [126].

**FIGURE 3:**
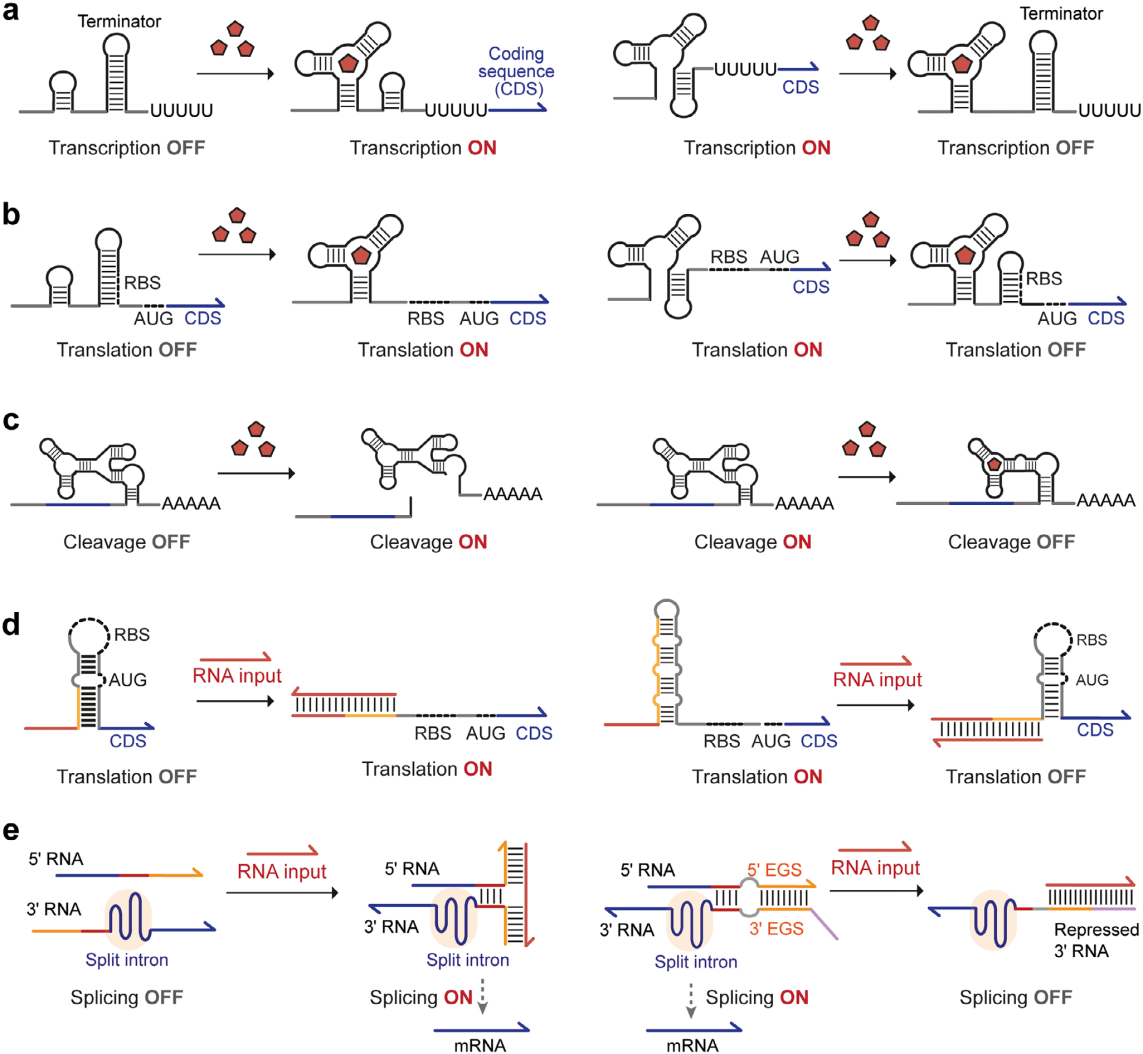
Synthetic biosensors based on RNA‐level regulation. (a) Riboswitches based on transcription termination control. The formation of terminator hairpin can be prevented (left panel) [121] or promoted (right panel) [131, 132] by ligand binding, activating or repressing target gene expression, respectively. (b) Riboswitches based on translation initiation control. The ribosome‐binding sites (RBSs) can be exposed (left panel) [133] or occluded (right panel) [134] by ligand binding, activating or repressing target gene expression, respectively. (c) Riboswitches based on self‐cleaving ribozymes. The ligand binding activates (left panel) [135] or represses (right panel) [65] the cleavage activities of the ribozymes. (d) Riboregulators based on translational control. The binding of input RNAs to the toehold domain exposes (left panel) [136] or sequesters (right panel) [137] the RBSs, activating or repressing gene translation. (e) Riboregulators based on self‐splicing group I introns. The assembly of split introns can be facilitated (left panel) [138] or disrupted (right panel) [139] by input RNAs, activating or repressing RNA splicing, respectively. CDS, coding sequence.

For transcription termination control, ligand binding induces a conformational change in the RNA that either promotes or prevents the formation of the terminator hairpin, terminating or initiating gene transcription (Figure 3a). A recent example is the fluoride‐responsive riboswitch, which folds co‐transcriptionally into two conformations, depending on the presence of fluoride [127]. The binding of fluoride stabilizes the aptamer structure and delays terminator nucleation for transcription readthrough. By contrast, in the absence of fluoride, the aptamer is destabilized and the terminator hairpin forms to trigger transcription termination. The fluoride‐responsive riboswitch has been leveraged to implement synthetic biosensors in the cell‐free system [128] and artificial cell [129]. More recently, a Co^2+^/Ni^2+^ riboswitch‐based biosensor, where metal ion binding promotes the formation of an anti‐terminator structure, has been engineered for environmental heavy metal monitoring [130].

For translation initiation control, ligand binding exposes or occludes the ribosome‐binding sites (RBSs) to regulate the downstream gene expression (Figure 3b). In a natural lysine‐responsive riboswitch from *E. coli*, the binding of lysine onto the aptamer domain not only inhibits translation initiation by sequestering RBS, but also exposes RNase E cleavage sites for mRNA degradation [134]. This lysine‐OFF riboswitch has been developed into lysine‐responsive biosensors in *Corynebacterium glutamicum*, to repress the competing pathways of lysine biosynthesis [140]. Subsequently, sequences between the aptamer domain and RBS were directly evolved to reverse the original lysine‐OFF riboswitch to lysine‐ON [133]. The lysine‐ON and ‐OFF riboswitches were applied to regulate lysine transport and suppress competing pathways, respectively, increasing the lysine production yield synergistically.

Besides engineering natural riboswitches, novel riboswitches could be designed by substituting the natural aptamer domains with de novo‐created aptamers and by developing novel expression platforms. Artificial aptamer sequences against virtually arbitrary target ligands could be selected using SELEX (Systematic Evolution of Ligands by EXponential enrichment). Adopting this technique, a dopamine‐binding RNA aptamer was selected in vitro [141] and assembled with a terminator‐based expression platform to devise a synthetic riboswitch for semi‐quantitative analysis of dopamine in human urine [142]. Likewise, an artificial‐cell‐based biosensor for histamine, a neurotransmitter, was engineered by utilizing SELEX to obtain histamine‐responsive RNA aptamers for integration with translation regulation [143]. Furthermore, the aptamer domains could be incorporated with novel expression platforms. Aptazymes, or ligand‐responsive ribozymes, can be constructed by fusing RNA aptamers with ribozymes (e.g., self‐cleaving hammerhead, hepatitis delta virus (HDV), and twister ribozymes) (Figure 3c). Recently, Fukunaga et al. integrated artificial aptamer selection with a circularly permuted pistol ribozyme to develop a mammalian biosensor for ASP2905, a small molecule for the treatment of Alzheimer's disease and schizophrenia [144]. Moreover, aptamers can be coupled to the ADAR (adenosine deaminase acting on RNA)‐based RNA editing system for detecting intracellular ATP and NF‐κB (nuclear factor‐kappa B) in mammalian cells [145].

Recent advancements in RNA structure prediction, thermodynamic modeling, and high‐throughput screening have accelerated the design of synthetic riboswitches. Automated computational tools have been developed for designing riboswitches that sense diverse chemicals and proteins [146, 147]. High‐throughput pipelines leveraging next‐generation sequencing were also established to screen for novel RNA aptamers in vivo [17, 148] and to optimize the communication modules between aptamers and expression platforms [149]. However, it remains challenging to identify RNA aptamers capable of sensing a specific molecule and transducing the signal to target gene expression in vivo.

#### 2.3.2 Riboregulators

Riboregulators are trans‐acting RNA elements that sense RNA inputs and dynamically reconfigure their RNA structures to control downstream gene expression. A typical riboregulator comprises a switch RNA that regulates target gene expression in cis and a trans‐acting RNA that binds to the switch RNA to modulate its conformation and activity. The highly predictable RNA base‐pairing mechanisms enable the de novo design of riboregulators to detect virtually any input RNA sequences, providing programmable platforms for developing RNA biosensors. To date, a wealth of riboregulators have been developed, including toehold switches [136], small transcription‐activating RNAs (STARs) [150], loop‐initiated RNA activators (LIRAs) [151], and split‐intron‐enabled trans‐splicing riboregulators (SENTRs) [139], enabling RNA‐responsive regulation of transcription, translation, and splicing.

As a classic riboregulator in prokaryotic systems, the toehold switch inhibits mRNA translation initiation by sequestering the RBS and start codon within a hairpin structure [136, 137]. Binding of input RNAs to the toehold region initiates the RNA strand displacement to open the hairpin structures to expose the RBS and start codon, activating the translation of reporter genes (Figure 3d). Toehold switches have been demonstrated to sense endogenous RyhB sRNA expression in *E. coli* [136], virus RNA fragments [7], and bacterial species‐specific mRNAs [23] in cell‐free systems freeze‐dried onto paper discs. The sensing specificity of toehold switches was enhanced by introducing an energy‐balancing region that distinguishes input RNAs with single‐nucleotide mutations and epitranscriptomic modifications (e.g., methylation) [152]. Moreover, the toehold switch has been extended to mammalian systems for sensing endogenous RNA levels [153]. Despite its tremendous success, toehold switch design still faces several drawbacks. For instance, the toehold switches add extra amino acids to the N‐terminus of the output protein, with unpredictable effects on the folding of output molecules. Moreover, the functionality of toehold switches relies on the host‐specific regulatory element (RBS), which affects their cross‐host portability.

To address these issues, a class of split‐intron‐enabled trans‐splicing riboregulators (SENTRs) [139] has been developed based on group I intron‐mediated RNA splicing. Group I introns are self‐splicing ribozymes that catalyze their own excision from precursor RNAs and seamlessly ligate the flanking exons to generate the mature mRNA. Group I introns are highly programmable and can be split at stem‐loop structures and reassembled for intron trans‐splicing once both intron halves are expressed [154]. In the SENTR design, split introns are inserted into the target mRNA to separate the mRNA into two RNA strands (5' RNA and 3' RNA) and rejoin them via RNA trans‐splicing, directed by the de‐novo‐designed external guide sequences (EGSs). SENTRs exhibit low leakage expression, wide dynamic ranges (>1000‐fold), and high predictability with machine learning. Moreover, SENTRs can sense intracellular mRNAs of bacterial antibiotic resistance genes, using EGS to recognize specific mRNA sequences for inhibition of RNA trans‐splicing activities (Figure 3e, right), process input RNA signals via ribocomputing, and transduce them into diverse outputs (mRNAs or non‐coding RNAs) [139]. Furthermore, RNA‐triggered intron trans‐splicing has also been achieved using the RENDR (Ribozyme‐Encoded Native RNA Detection) platform [138], which fuses each split intron half to RNA guide sequences designed to interact with the input RNA. Upon input RNA presence, the guide sequences hybridize to colocalize split intron halves, which assemble into a functional intron complex for splicing to produce the mRNA encoding output proteins (Figure 3e, left). Similar designs are employed to detect bacterial antibiotic resistance gene expression in *E. coli* [138], report DNA transfer in the soil microbiome [155], and visualize endogenous RNAs in plants [156, 157].

### 2.4 Biosensors Based on CRISPR/Cas Systems

The CRISPR (Clustered Regularly Interspaced Short Palindromic Repeats)/Cas systems, which originate in bacterial adaptive immunity, have emerged as powerful tools for synthetic gene regulation and nucleic acid sensing due to their excellent modularity, programmability, and specificity [158, 159]. In this section, we focus on the genetically encoded CRISPR‐based biosensors for in vivo RNA and DNA detection, with emphasis on several recently developed design strategies (i.e., tracrRNA reprogramming, RNA‐induced protein cleavage, and strand displacement of gRNA) and applications (i.e., biosensing of single‐nucleotide polymorphisms).

#### 2.4.1 TracrRNA Reprogramming

**FIGURE 4:**
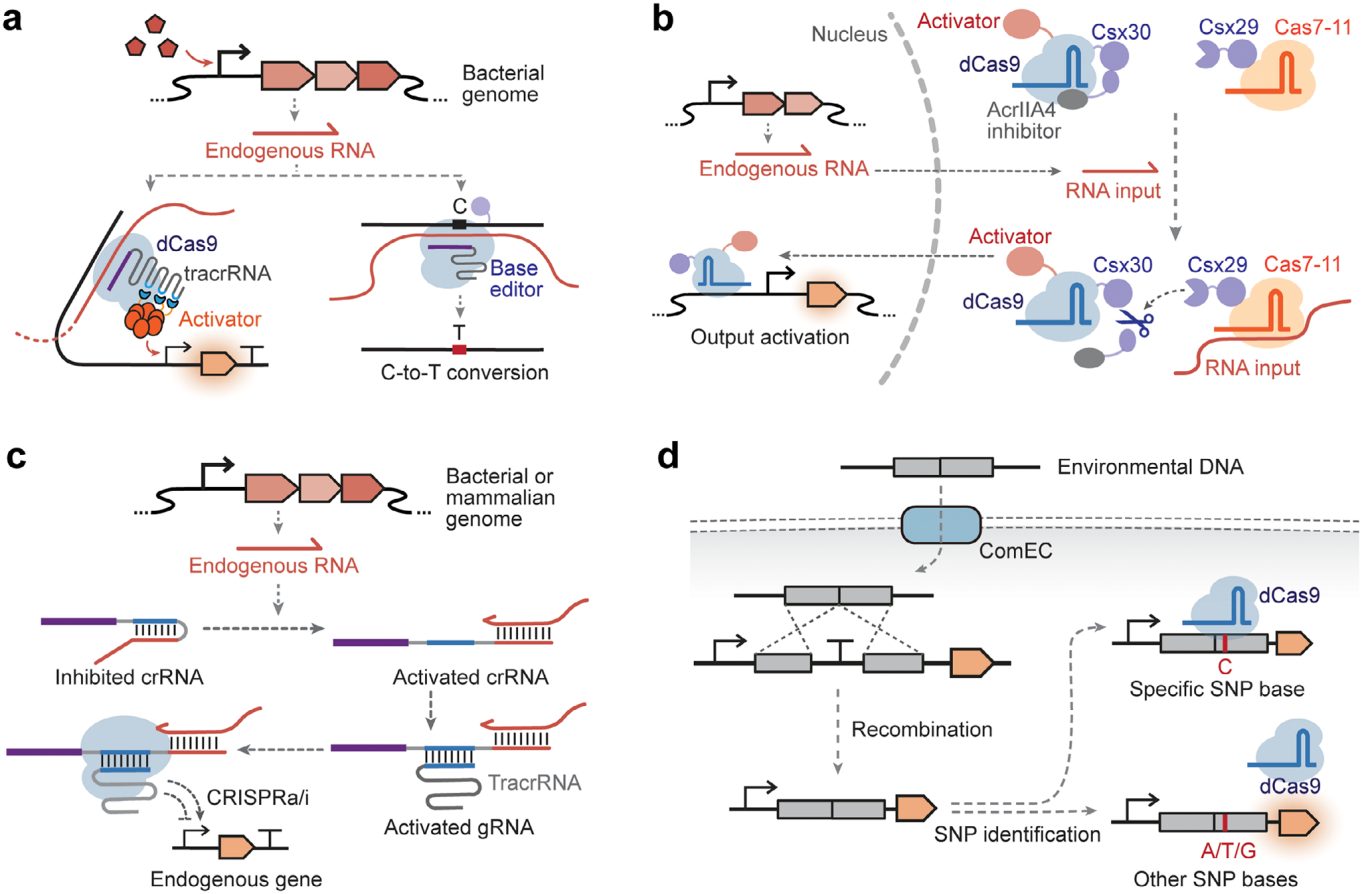
Biosensors based on CRISPR/Cas systems. (a) Biosensors based on reprogramming tracrRNA‐crRNA pairing. TracrRNAs can be reprogrammed to hijack endogenous RNAs as crRNAs, to form a functional gRNA complex, turning on the eukaryote‐like CRISPR activation (CRISPRa) system (left panel) [16, 160] or activating a base editor to record the stimuli (right panel) [162]. (b) Biosensors based on RNA‐induced protein cleavage [163], in which dCas9 was N‐terminally fused to Csx30 and the dCas9 inhibitor AcrIIA4. Recognition of the target RNA by Cas7‐11‐Csx29 leads to cleavage of Csx30, releasing the dCas9‐VPR‐gRNA complex from the AcrIIA4 inhibitor and activating the output gene expression. (c) Biosensors based on strand‐displacement of gRNA. The binding of endogenous RNA to the toehold domain releases the repeat region for crRNA‐tracrRNA assembly and activates the CRISPR systems to regulate endogenous gene expression in *E. coli* and mammalian cells. [164] (d) DNA Biosensors leveraging CRISPR/Cas systems for single‐nucleotide polymorphism (SNP) recognition. [165] Human DNA is transported into *Bacillus subtilis* and integrated into the bacterial genome via recombination, and the sgRNA then guides dCas9 to the SNP region of the target DNA. If the sgRNA matches the SNP, dCas9 will block transcription of the target gene. By contrast, the mismatch between sgRNA and the SNP region will lead to gene activation.

In natural type II CRISPR/Cas9 systems, the guide RNA (gRNA) is composed of the CRISPR RNA (crRNA) and the trans‐activating CRISPR RNA (tracrRNA). The crRNA consists of a spacer domain for specific DNA sequence recognition and a repeat region for hybridization with tracrRNA. The tracrRNA binds to crRNA and Cas9 to form an active gRNA‐Cas9 complex. Recent studies have revealed that the crRNA‐tracrRNA pairing region is highly programmable, allowing for any sequence substitution and enabling the development of RNA sensors. Liu et al. [16] reprogrammed tracrRNAs to hijack endogenous RNAs as crRNAs, to form a functional gRNA complex, turning on the eukaryote‐like CRISPR activation (CRISPRa) system [160] and thus, triggering output gene expression. This system has been utilized to assess endogenous mRNAs and sRNAs transcribed from environmental‐responsive genes in *E. coli*, serving as biosensors for detecting hydrogen peroxide, arsenic, zinc, lead, and copper (Figure 4a). This scheme was then extended to an in vitro transcription‐based biosensor, AGATHA, for detecting SARS‐CoV‐2 RNA fragments within 10 min [16]. In the AGATHA biosensor, the reprogrammed hijacked the RNA inputs to activate the Cas9, cleaving off the anti‐Broccoli tail in the target DNA. This Cas9‐mediated cleavage modifies the in vitro‐transcribed reporter RNA sequence, enabling the correct folding of the Broccoli aptamer and producing a fluorescent output signal. Similarly, two other RNA sensors, LEOPARD [161] and TIGER [162], have been developed by reprogramming tracrRNAs for RNA detection with single‐base resolution in vitro and recording endogenous RNA expression in vivo, respectively.

Compared with dCas13/dCas9‐based in vivo RNA‐sensing platforms, tracrRNA reprogramming decouples target RNA sequences from gRNA spacers, thereby enabling linking RNA detection events to diverse CRISPR functions. Compared with in vitro CRISPR diagnostic platforms based on Cas12a/Cas13 that search a single target to elicit non‐specific DNA/RNA cleavage of fluorescent reporters, tracrRNA reprogramming redirects different RNA targets to distinct DNA sequences, offering a scalable, multiplexed detection platform for simultaneous, parallel detection of multiple RNA biomarkers in a single reaction.

#### 2.4.2 RNA‐Induced Protein Cleavage

In type III‐E CRISPR systems, the effector Cas7‐11 binds to the crRNA and protease Csx29. Target RNA recognition by crRNA‐Cas7‐11 triggers a conformation change in Csx29, activating its proteolytic activity to cleave its substrate Csx30. Leveraging this mechanism, Strecker et al. [163] developed a mammalian RNA sensor by anchoring Cre recombinase to the cell membrane via a Csx30‐derived linker, which could be cleaved by the crRNA‐Cas7‐11‐Csx30 complex upon target RNA binding. Cre recombinase is then translocated to the nucleus to activate expression of the output gene. In a following study, Zhang et al. [166] developed an RNA‐IN/RNA‐OUT biosensing circuit by coupling Cas7‐11‐Csx29 with a dCas9‐VPR‐based CRISPRa system. In this circuit, dCas9 was N‐terminally fused to Csx30 and the dCas9 inhibitor AcrIIA4. Recognition of the target RNA by Cas7‐11‐Csx29 leads to cleavage of Csx30, releasing the dCas9‐VPR‐gRNA complex from the AcrIIA4 inhibitor and activating the output gene expression (Figure 4b). The RNA‐IN/RNA‐OUT circuit was demonstrated to rewire endogenous gene networks, dynamically monitor cell‐state transitions during cell differentiation and trans‐differentiations, and selectively kill cancer cells.

#### 2.4.3 Strand‐Displacement of gRNA

The synthetic RNA‐sensing function of the CRISPR system can also be created by designing switchable gRNAs that exploit toehold‐mediated strand‐displacement reactions. For instance, in the toehold‐gated gRNA (thgRNA) [167], the spacer sequences are sequestered by an artificially designed stem‐loop structure in the absence of a target RNA, but are re‐exposed via strand displacement upon target RNA presence, thereby restoring CRISPR function for gene expression regulation. The thgRNA can be selectively activated by intracellular mRNAs and endogenous sRNAs for conditional gene knock‐out in *E. coli*. However, spacer sequences for thgRNAs need to be designed according to the target input sequences, making it challenging to regulate endogenous gene expression. To establish regulatory linkages between endogenous gene expression, the toehold domain is inserted into the repeat region of the gRNA to modulate the pairing between crRNA and tracrRNA [164]. Upon sensing endogenous RNAs, the toehold region initiates branch migration, releasing the repeat domain for crRNA‐tracrRNA assembly and activating the CRISPR systems to regulate endogenous gene expression in *E. coli* and mammalian cells (Figure 4c).

#### 2.4.4 CRISPR/Cas Systems for SNP Recognition

The high specificity of CRISPR‐Cas systems enables the recognition of DNA sequences containing SNPs (single‐nucleotide polymorphisms). Therefore, the incorporation of CRISPR systems can enhance the sequence specificity of other biosensing systems. For instance, Nou et al. [165] applied CRISPR inference to distinguish single‐nucleotide variants of environmental DNA taken up by the bacterial cell. In this DNA biosensor, human DNA is transported into *Bacillus subtilis* and integrated into the bacterial genome via recombination, and the sgRNA then guides dCas9 to the SNP region of the target DNA (Figure 4d). If the sgRNA matches the SNP, dCas9 will block transcription of the target gene. A single mismatch between the sgRNA and the SNP region will reduce dCas9's affinity for the target DNA, leading to transcription elongation and gene activation. In another study, CRISPR/Cas9‐mediated cleavage was integrated with toehold switches to develop cell‐free biosensors capable of discriminating between American‐ and African‐lineages of Zika virus with single‐base differences in the CRISPR‐targeting region [7].

## 3 Enhancing Biosensing Performance by Genetic Circuit Design

The performances of synthetic biosensors can be evaluated in five dimensions: sensitivity, specificity, speed, stability, and biosafety (Table 2). In this section, we review the design principles for deploying a high‐performing biosensor through genetic circuit design, as well as the enabling tools and strategies to enhance or alter specific characteristics of biosensors' performance.

**TABLE 2:** Performance evaluation of representative biosensing mechanisms.

Biosensing mechanism	Advantage	Challenge
Allosteric transcription factor (aTF)	Sensitivity: Systematic optimization methods enabled high dynamic ranges, low detection limits or thresholds, and tunable operational ranges. Specificity: ATFs can detect a broad range of chemicals and their various analogs with high specificity, which can be further improved via established methods such as directed evolution. Stability: ATFs can be interfaced with genetic controllers for enhancing biosensing stability. Biosafety: ATFs are well compatible with cell‐free expression systems (CFESs) or artificial cells, allowing for detection without the use of genetically modified organisms (GMOs).	Speed: ATF‐based sensors typically require gene transcription and translation to produce reporter proteins, resulting in relatively slow operation.
Two‐component system (TCS)	Sensitivity: Multiple sensory components, such as histidine kinases and response regulators, serve as tuning knobs for sensitivity and dynamic ranges. Specificity: TCSs are highly specific for their native target ligands or physical signals, but the limited plasticity of HKs constrained the alteration and expansion of target specificity of TCS sensors.	Speed: Multi‐step signal transduction leads to slow responses. Stability: Potential cross‐interactions with endogenous pathways reduce performance robustness and cross‐species portability. Biosafety: TCSs are most often employed in whole‐cell bacterial biosensors, which can pose potential biosafety risks.
Riboswitch	Speed: RNA‐level regulation operates at fast time‐scales. Biosafety: Riboswitches are well compatible with CFESs or artificial cells, enabling detection without GMOs.	Sensitivity: Dynamic ranges and sensitivity are limited for sensing intracellular ligands. Specificity: Riboswitches possess relatively low inherent specificity, but offer excellent evolvability, allowing high‐throughput selection of highly specific RNA aptamers. Stability: Riboswitches exhibit context‐dependent behavior and are susceptible to disturbances in cellular states and extracellular environments.
Riboregulator	Specificity: Riboregulators show high specificity, enabling discrimination of target nucleic acids at single‐base resolution. Biosafety: Riboregulators are compatible with CFESs for in vitro detection of nucleic acids. Speed: RNA‐level regulation operates at fast time‐scales.	Sensitivity: Riboregulators that sense intracellular RNAs typically show limited sensitivity, and an isothermal nucleic acid amplification step is required to enhance in vitro RNA detection sensitivity. Stability: Riboregulators exhibit context‐dependent behavior, with detection performances largely influenced by surrounding genetic sequences.
CRISPR/Cas system	Specificity: The high specificity of CRISPR/Cas systems allows discrimination of target nucleic acids at single‐base resolution. Biosafety: CRISPR diagnostic platforms for in vitro nucleic acid detection are well‐established and require no GMOs.	Sensitivity: CRISPR/Cas systems possess high sensitivity for in vitro nucleic acid detection but relatively low dynamic ranges and sensitivity for in vivo RNA sensing. Speed: RNA/DNA detection in vivo is slow. Stability: Cas proteins are often burdensome to express, affecting long‐term stability in vivo.

### 3.1 Tuning Sensing Sensitivity

Biosensors are expected to detect low concentrations of target ligands and produce significant output signals in response to small changes in target ligand concentrations in real‐world applications, such as environmental monitoring, food safety assessment, and disease diagnosis. To function effectively, biosensors need to be highly sensitive, with low detection limits and thresholds, minimal leakiness, and a wide dynamic range.

**FIGURE 5:**
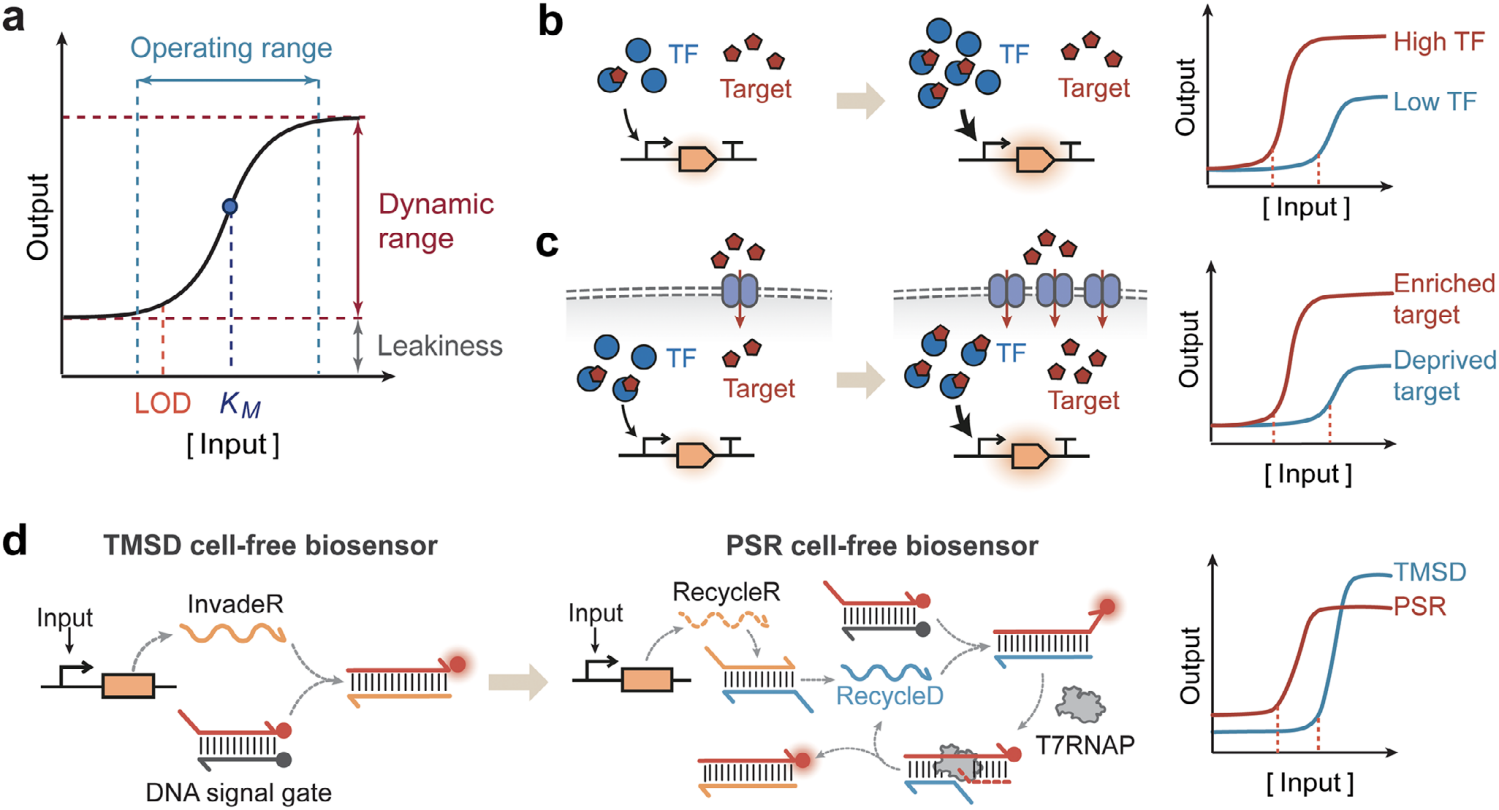
Dose‐response curve (a) and strategies to tune detection limits of synthetic biosensors (b–d). (a) Biosensors' sensitivity can be systematically evaluated using the response curve with essential metrics comprising limit of detection (LOD), detection threshold (*K_M_*), operating range, dynamic range, and leakiness. (b) Tuning concentrations of ligand‐responsive receptors can lower the LOD and *K_M_*. [168] (c) Tuning the expression levels of transporters can enrich intracellular target ligands and enhance sensor sensitivity. [45] (d) Polymerase strand recycling (PSR) circuit [27] leverages the T7 RNA polymerase (T7RNAP) off‐target transcription activities in cell‐free systems, to recycle the nucleic acid inputs and reduce the LOD of the toehold‐mediated strand displacement (TMSD) biosensors [169].

Biosensors' sensitivity can be systematically evaluated using the response curve (Figure 5a), with essential metrics comprising: (i) Limit of detection (LOD): the minimum input levels required to elicit a significant output signal; (ii) Detection threshold (*K_M_*): the target concentration at which the biosensor's response is at the midpoint between the basal leakiness level and the maximum output magnitude; (iii) Operating range: the detection window within which the concentrations of a input target can be quantified through a change in output signals; (iv) Dynamic range: the ratio of the maximum output magnitude to the basal background levels; (v) Leakiness: the baseline output level of the biosensor in the absence of the input signal.

In the following sections, we review the design principles, strategies, and enabling tools for rationally tuning a specific parameter in the response curve of a synthetic biosensor. Design principles for synthetic biosensors have primarily been developed in bacterial biosensors but can also be applied to eukaryotic and cell‐free biosensors, as discussed throughout the review.

#### 3.1.1 Lowering the Detection Limit and Threshold

The limit of detection and detection threshold determine the minimal ligand concentrations that biosensors can detect, which can be reduced by altering the ligand receptor concentrations, increasing the local concentrations of target ligands, and selecting an appropriate output reporter. It is noteworthy that the detection thresholds have been referred to in several forms, such as *K_1/2_* [113], half‐maximal effective concentration EC_50_ [14], and Hill constant *K_M_* [5], which we adopted in this review.

The concentrations of ligand‐responsive receptors play a vital role in deciding the detection limits and thresholds. Such ligand‐responsive receptors include aTFs, response regulators (RRs) in two‐component systems (TCSs), and G‐protein coupled receptors (GPCRs), as assessed in bacterial, eukaryotic, and cell‐free biosensors. For the aTF‐based biosensors, reducing the intracellular densities of transcription repressors or increasing the expression levels of transcription activators can significantly reduce the detection thresholds of bacterial biosensors without increasing leakiness (Figure 5b) [168]. Studies on *E. coli* biosensors leveraging constitutive promoters with varying strengths (promoters J23117 to J23101 on a medium‐copy plasmid pSB3K3) to express transcription repressors (TetR, ArsR, and MerR) have revealed that weaker promoter strengths lead to higher sensitivity and output magnitude [5, 168]. By contrast, using stronger promoter (J23101) to express transcription activators LuxR lowered *K_M_* and increased output levels, without introducing notable noise or detrimental effects on bacterial growth. Similarly, reducing the amount of transcription repressors in cell‐free systems sensitized the aTF‐based cell‐free biosensors for copper and lead [6]. For TCS‐based biosensors, the detection thresholds are decided by the relative concentrations of phosphorylated RRs. Therefore, mutations that improve HK kinase or reduce phosphatase activity can be used to rationally lower TCS detection thresholds, as demonstrated in the *B. subtilis* TCS sensor for nitrate and the *E. coli* TCS sensors for aspartate, tetrathionate, and thiosulfate [113]. For GPCR‐based biosensors, elevating the expression levels of the transmembrane GPCR ste20 has been shown to boost the dose‐response sensitivity of the yeast biosensor for α‐factor [4].

Enriching the target ligands inside the detection systems can also elevate the sensors' sensitivity. For cell‐based biosensors, accumulation of intracellular target ligands can be achieved by enhancing the expression of import machinery and by deleting or inhibiting efflux pumps (Figure 5c). For example, Chen et al. lowered the LODs of bacterial sensors for uric acid and lactate by exogenously overexpressing transporters to enhance ligand uptake [45]. In cell‐free biosensors for nucleic acid detection, the target ligands can be enriched using nucleic acid amplification techniques such as rolling circle amplification (RCA) [170] for DNA substrates and nucleic acid sequence‐based amplification (NASBA) [7] for RNA substrates. In the case of toehold‐switch‐based cell‐free sensors for detecting Zika RNA, incorporation of NASBA reduced the LOD from 30 nM to 3 fM [7]. More recently, a polymerase strand recycling (PSR) circuit was developed based on T7 RNA polymerase off‐target transcription activities in cell‐free systems, reducing the LOD of zinc and tetracycline sensors by 10‐fold (Figure 5d) [27].

The choice of output reporters also significantly impacts the sensing sensitivity. A systematic evaluation of eight reporters, including fluorescent, colorimetric, and bioluminescent reporters in both cell‐based and cell‐free biosensors, reveals differences in their contributions to sensing performance [171]. Interestingly, the bioluminescent reporters exhibited much lower LODs (at least 3 orders of magnitude lower) than fluorescent reporters in cell‐based biosensors for mercury and 3OC_6_HSL, whereas the fluorescent reporters provided the best LODs in the cell‐free sensors for detecting the same ligands. Such differences in fluorescent reporter behavior might result from contextual factors, such as the lack of cell membranes and metabolic buffering in the cell‐free expression system (CFES), variations in post‐translational modification levels, different background fluorescence, and the use of a reporter variant (deGFP) that is more translatable in CFES.

#### 3.1.2 Modulating Operating Range

Operating range refers to the range of input signals that biosensors can respond to. Biosensors with a narrow operating range are demanded to generate a digital‐like response in applications such as on‐site detection of food toxins or environmental pollutants. By contrast, biosensors with analog responses and wide operating ranges can be employed to precisely determine the concentrations of input ligands with applications such as monitoring product yields of cell factories and biomarker‐responsive release of therapeutic biomolecules.

Recombinase‐based comparators can transform biosensors' graded analog responses into digital‐like responses with narrow operating ranges. These genetic comparators comprise a ligand‐responsive promoter for driving recombinase expression and determining the threshold for comparator activation and a digitalizer module for recombinase‐mediated inversion of the target DNA orientation to activate output gene expression [172]. Incorporation of genetic comparators to a hydrogen peroxide biosensor narrowed the operating range from 0.1–100 µM to 1–6 µM in *E. coli*. Notably, inhibitory protein sequestration [173] and cooperative regulatory assemblies [18] have also been shown to enhance the ultrasensitivity of genetic circuits, but their applications in biosensors have yet to be explored.

Cell population‐based strategies have also been developed for transitioning operating ranges. The extension of operation ranges is achieved by mixing cell‐based biosensors with different sensitivities to a target ligand [4]. As a result, the mixed cell population will exhibit the average output levels of these biosensors at various input levels, linearizing the original steep response curves. By contrast, narrowing the operating range is achieved through cell‐to‐cell communication [4]. In the two‐cell system, the first yeast cell detects the input signal and secretes α‐factor, which can be responded to by the second cell for output activation. At the same time, the second cell constitutively secretes a protease, Bar1, to degrade α‐factor below the threshold levels, digitalizing the response curve.

Operating ranges can also be modulated through incorporating genetic circuit topologies. Negative feedback loops (NFLs), for instance, have been demonstrated to increase sensor operational range by linearizing the input‐output response, as exemplified by AraC‐based autorepression in *E. coli* [174]. More recently, synthetic NFL was implemented in mammalian cells using a CRISPR activation device to express anti‐CRISPR protein ACRIIA4 [175]. The NFL incorporation into the mammalian copper biosensor significantly improved linearity and operating range, though at the cost of elevated leakiness and decreased dynamic range. Positive feedback loops (PFLs) can either narrow or expand the operating range, depending on the specific circuit architecture. Notably, two PFLs have been incorporated into a single yeast biosensing circuit that encodes a transmembrane receptor and a transcription factor, transforming the original graded response into a binary one with a 10‐fold narrower operating range [176]. In this two‐PFL topology, extracellular ligands bind to the receptor on the cell surface, activating the intracellular transcription factor, which in turn upregulates its own expression as well as the receptor expression. For broadening operating ranges, self‐activating aTFs expressed on low‐copy plasmids were utilized to activate reporter expression on high‐copy plasmids, yielding logarithmically linear transfer functions [177].

#### 3.1.3 Increasing Dynamic Range

High dynamic range is crucial for achieving reliable, sensitive detection in biosensors. Dynamic range optimization can be realized by minimizing basal leakage and increasing the maximal output signal amplitude. The strategies for dynamic range optimization include promoter and RBS engineering, as well as the implementation of genetic amplifiers.

**FIGURE 6:**
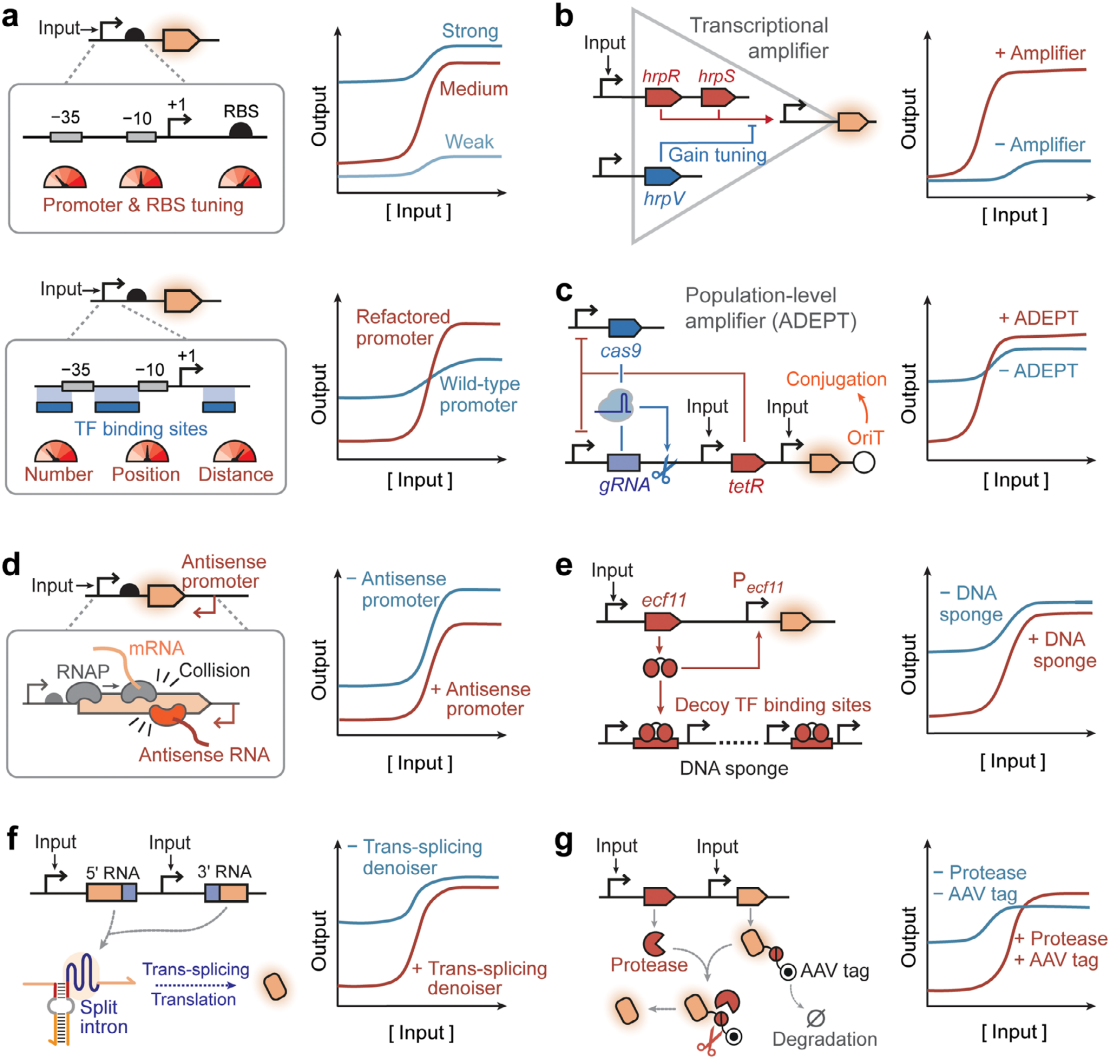
Strategies to increase the dynamic ranges (a–c) and minimize leakiness of synthetic biosensors (d–g). (a) Promoter and RBS engineering can be utilized to optimize dynamic range. Upper panel, tuning the promoter and RBS strengths. [178] Lower panel, refactoring promoters by altering the number and position of transcription factor (TF) binding sites, as well as distances between multiple TF binding sites. [179] (b) Transcriptional amplifier leverages ultrasensitive transcription activators to boost dynamic ranges. [5, 70] (c) Population‐level amplifier integrates plasmid loss rate control mediated by CRISPR/Cas9 system, gene transfer regulation based on F‐conjugation, and fitness control by antibiotic selection. [118] (d) Antisense transcription suppresses leakiness by causing collision between the actively transcribing RNAPs, as well as producing antisense RNAs for translation inference. [12, 119] (e) DNA sponge contains decoy TF‐binding sites to compete with the output promoter to sequester intracellular TFs. [180] (f) Trans‐splicing denoiser comprises 5' RNA and 3' RNA, which are separately transcribed by output promoters and splice into intact mRNAs encoding output proteins. [45, 139] (g) Protease‐regulated protein degradation employs ligand‐responsive protease expression to cleave degradation tags from the output proteins, rescuing them from protein degradation. [5]

Promoter engineering can modulate interactions among transcription factors (TFs), RNA polymerases (RNAPs), and output promoters, thereby altering both leakiness and maximal output levels. On the one hand, the binding equilibrium between the RNAP and the promoter can be tuned by varying the sequences at the −10 and −35 sites, thereby altering the dynamic range of the ligand‐responsive promoters (Figure 6a, upper panel). Guided by this principle, Chen et al. [178] characterized a library of sequences and combinations of −10 and −35 sites, to engineer optimal promoters with the highest inducibility and lowest leakiness. Promoters with affinities for RNAP that are too strong or too weak can yield limited dynamic ranges, and high transcription rates are usually associated with a heavy metabolic burden imposed on host cells, which affects host growth and compromises long‐term sensor stability. On the other hand, the strength of TF‐promoter interactions could be fine‐tuned by altering the numbers, positions, and distances of TF‐binding sites (TFBSs) (Figure 6a, lower panel). An extra TFBS can be inserted into the original promoter, creating a roadblock that reduces leakiness without sacrificing maximal output [5]. Moreover, TFBS positions and distances between multiple TFBSs can be further optimized to maximize biosensors' dynamic ranges [5, 6]. For instance, transcription repressor‐mediated inhibition is most potent with the TFBS inserted at the core region between the −10 and −35 sites, followed by those at proximal and distal sites, downstream and upstream of the core region, respectively. Moving the wild‐type AsrR binding site from the proximal site to the core region yielded a refactored promoter with simultaneously reduced leakage and improved signal output [179].

Translation rates of output mRNAs, determined by RBS choices, affect leakiness and maximal outputs most directly. Strong RBSs increase the maximal outputs but lead to leaky basal expression, while weak RBSs exhibit low leakiness but poor induction (Figure 6a). RBS strengths also indirectly affect sensor dynamic ranges through host‐circuit interactions; that is, strong RBSs may lead to resource competition between synthetic circuits and endogenous host circuits at the translational level, thereby interfering with host cellular processes and, in turn, affecting sensor performance [181, 182]. Therefore, titrating RBS strengths is important for optimizing dynamic range. Synthetic RBSs can be either rationally designed via computational tools (e.g., RBS calculator) [183] or selected from a well‐characterized, standard part library. Recently, Buson et al. established BiopartsDB [1], a small‐scale, curated online database with handpicked genetic parts from high‐quality publications, providing a reliable platform for synthetic biologists to select genetic parts for biosensor development.

In addition to part engineering, genetic circuit‐based strategies have been developed to amplify dynamic ranges in a modular, composable manner. Transcriptional amplifiers can be implemented by rewiring the output of ligand‐responsive biosensors (e.g., aTF‐regulated promoters [70] and riboswitches [184]) to drive the expression of ultrasensitive transcription activators, such as HrpRS in bacteria [70] and the tetracycline transcriptional activator (rTA) in mammalian cells [185]. For the HrpRS‐based amplifier, an additional module encoding the inhibitor HrpV can regulate HrpRS activity, enabling controllable tuning of amplification gains (Figure 6b) [70]. The dynamic range amplification performance can be further boosted by connecting multiple transcriptional amplifiers [5]. Genetic amplifiers can also be engineered with plasmid copy number control, quorum sensing, and gene transfer [186, 187]. The most noticeable example is ADEPT, a population‐level signal amplifier integrating plasmid loss rate control mediated by the CRISPR/Cas9 system, gene transfer regulation based on F‐conjugation, and fitness control by antibiotic selection [118]. In the absence of input signals, the copy number of the target plasmids encoding output genes is maintained at a low level due to CRISPR/Cas9 cleavage, thereby suppressing the potential leakage expression. Upon ligand detection, the expression of gRNA and Cas9 is inhibited, and the target plasmid abundance is enriched through F‐conjugation‐mediated gene transfer and positive selection using antibiotics, to amplify the output signals (Figure 6c).

Genetic circuit topologies were also devised to improve dynamic ranges by boosting output magnitudes and reducing leakiness. Positive feedback loops (PFLs) based on transcription activator LuxR have been leveraged to amplify biosensor responses to cadmium and aspartate, where the ligand induces expression of LuxR to activate output gene expression and enhance its own transcription [188, 189]. Other topologies, such as coherent feedforward loops, enhance sensitivity and dynamic range by reducing system leakiness, as discussed in the following subsection.

#### 3.1.4 Minimizing Leakiness

Suppressing basal leaky expression is a critical step in enhancing the detection sensitivity of synthetic biosensors. So far, systematic strategies have been developed to reduce leakiness at all regulatory levels of gene expression.

At the transcriptional level, the leakiness from output promoters can be reduced through antisense transcription [119] or DNA sponge titration [180]. Antisense transcription can suppress the transcription activity by causing collision between the actively transcribing RNAPs, as well as producing antisense RNAs for translation inference (Figure 6d) [119]. DNA sponge contains multiple copies of DNA‐binding sites or cognate promoters of the decoyed TFs, which compete with the output promoter to sequester intracellular TFs (Figure 6e) [180]. Introducing DNA sponges into biosensors has been shown to reduce the leakiness by over 20‐fold and concurrently mitigates the metabolic burden caused by TF overexpression.

At the post‐transcriptional level, a trans‐splicing denoiser circuit [45] was designed based on split‐intron‐mediated trans‐splicing riboregulators [139], where the 5' RNA and 3' RNA are separately transcribed by output promoters and splice into intact mRNAs encoding output proteins (Figure 6f). This trans‐splicing denoiser leverages the requirement for a relatively high abundance of RNA substrates by intron splicing to suppress leakiness. Similar architectures have also been implemented by toehold switches [190] and the suppressor tRNA [191], which enables conditional translational readthrough of stop codons in target mRNAs.

At the post‐translational level, basal output protein levels can be reduced by protein splicing and degradation. Splitting highly active proteins (e.g., β‐lactamase, TetR, and ECF20) and reconstituting them by split intein‐mediated protein trans‐splicing can suppress their background activities [107]. The cooperation between protease cleavage and protein degradation provides another seminal example of leakiness reduction [5], in which an input signal not only activates output gene expression but also induces protease expression to cleave degradation tags from the output proteins, rescuing them from protein degradation (Figure 6g).

Despite various regulatory modalities having been developed to reduce leakiness, many of them also compromise the maximal expression level and lead to only moderate improvement in dynamic ranges. Therefore, such regulatory modalities need to be arranged in certain genetic circuit topologies to mitigate leakiness while maximizing induction magnitude, including coherent feedforward loop (CFFL) and mutual inhibition (MI) topologies, which both comprise three regulatory components, X, Y, and Z. In CFFL, the output Z is repressed by Y in the absence of input signals, thereby exhibiting minimal leakiness. The input X directly triggers the output Z and inhibits Y to derepress Z [192, 193]. In MI topology, the input X activates the output Z, while Z and Y inhibit each other [192, 194]. Genetic circuits encapsulating these topologies have been implemented using the abovementioned modalities (e.g., antisense transcription and protein degradation), demonstrating elimination of basal leakiness and tremendous amplification of dynamic ranges (to over 1000‐fold) in bacteria and mammalian cells [192, 194]. The sensor performances can be further improved through integration of CFFL, MI, or NFL into even more complex topologies such as coherent inhibitory loops that combine the advantages of all circuits [175, 192].

### 3.2 Enhancing Sensing Specificity

Natural biosensing mechanisms often exhibit substrate promiscuity, responding to non‐target ligands and generating misleading false‐positive signals. Strategies to improve sensing specificity include manipulating biorecognition elements by directed evolution and implementing genetic logic circuits.

**FIGURE 7:**
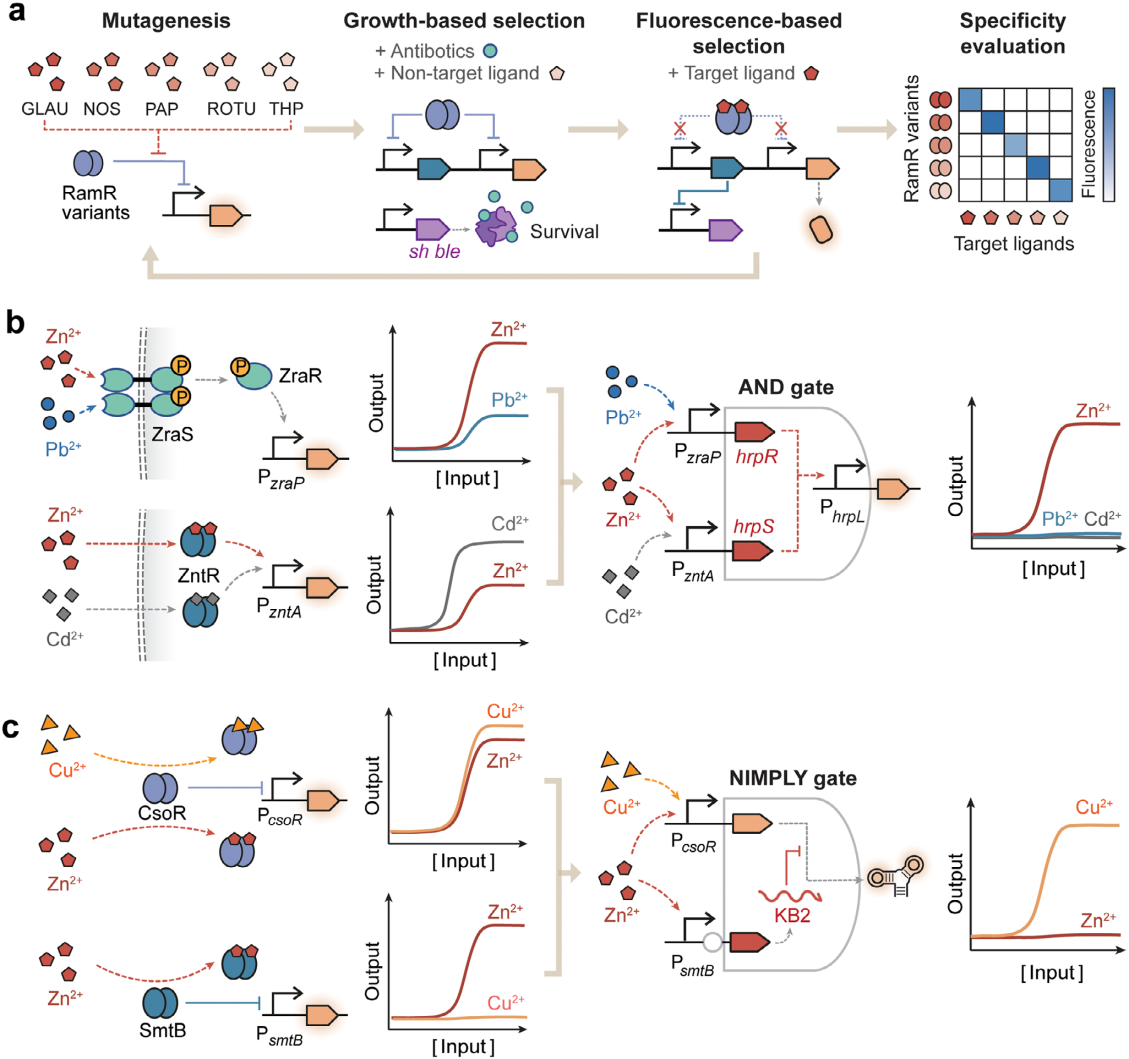
Improving biosensing specificity by directed evolution (a) and genetic logic circuits (b,c). (a) Direct evolution pipeline integrates growth selection and fluorescence screening for enhancing biosensing specificity. [14] The RamR variants repress the expression of a fluorescent protein and activate the expression of the antibiotic‐resistance gene *sh ble* via a repression cascade. For growth‐based negative selection, antibiotics and non‐target ligands are added to the bacterial culture to select against leaky or non‐specific RamR variants. For fluorescence‐based positive selection, target ligands are added to induce expression of output proteins. (b) The genetic AND gate couples two non‐specific biosensors, ZraR (responsive to Zn^2+^ or Pb^2+^) and ZntR (responsive to Zn^2+^ or Cd^2+^), to produce a specific response to Zn^2+^. [35] (c) The genetic NIMPLY gate couples a non‐specific biosensor, CsoR (responsive to Cu^2+^ or Zn^2+^), with SmtB, a Zn^2+^‐specific biosensor that expresses an inhibitory RNA KB2 to repress the output signal in the presence of Zn^2+^. [6]

The specificity of biorecognition elements (e.g., ligand‐binding domains in allosteric transcription factors and aptamer domains in riboswitches) can be improved through directed evolution. To enable rapid biosensor evolution, a genetic circuit named SELIS [14] was designed to combine growth‐based selection and fluorescence‐based screening, in which the aTF RamR variants repress the expression of a fluorescent protein and activate the expression of the antibiotic‐resistance gene *sh ble* via a repression cascade (Figure 7a). For growth‐based negative selection, antibiotics and non‐target ligands are added to the bacterial culture to select against leaky or non‐specific RamR variants. For fluorescence‐based positive selection, target ligands are added to induce expression of output proteins. This SELIS circuit was demonstrated to evolve RamR into five highly sensitive and specific biosensors for five distinct therapeutic alkaloids. More recently, Sensor‐seq, a high‐throughput platform based on next‐GENERATION sequencing, has been developed to enhance biosensors' specificity toward their cognate ligands or to alter their specificity toward new ligands [195]. Leveraging this platform, the authors identified TtgR variants with improved specificity for naringenin and phloretin, and novel sensing capabilities for six non‐native ligands.

Logic circuits assimilate the signals from non‐specific biosensors and selectively produce a response to target ligands. A genetic AND gate was used to couple two non‐specific biosensors, ZraR (responsive to zinc or lead) and ZntR (responsive to zinc or cadmium) (Figure 7b). The AND gate can generate an output signal only when both biosensors are activated, thereby enabling specific detection of zinc [35]. Moreover, a genetic NIMPLY gate was used to reduce the crosstalk between cell‐free biosensors and the non‐target ligands [6]. The NIMPLY gate contains two biosensors: CosR, which expresses the fluorescent aptamer 3WJdB in response to copper or zinc, and SmtB, a zinc‐specific biosensor that expresses an inhibitory RNA KB2 to repress the 3WJdB signal in the presence of zinc (Figure 7c).

### 3.3 Increasing Sensing Speed

Current genetic circuit‐based biosensors face major challenges in achieving fast response. Compared to their electronic or chemical counterparts capable of real‐time detection, biosensors typically require gene transcription and translation to produce reporter proteins, taking 30 minutes to several hours to generate significant output signals. Recent advances in developing novel sensing and output mechanisms have proposed solutions to this challenge.

**FIGURE 8:**
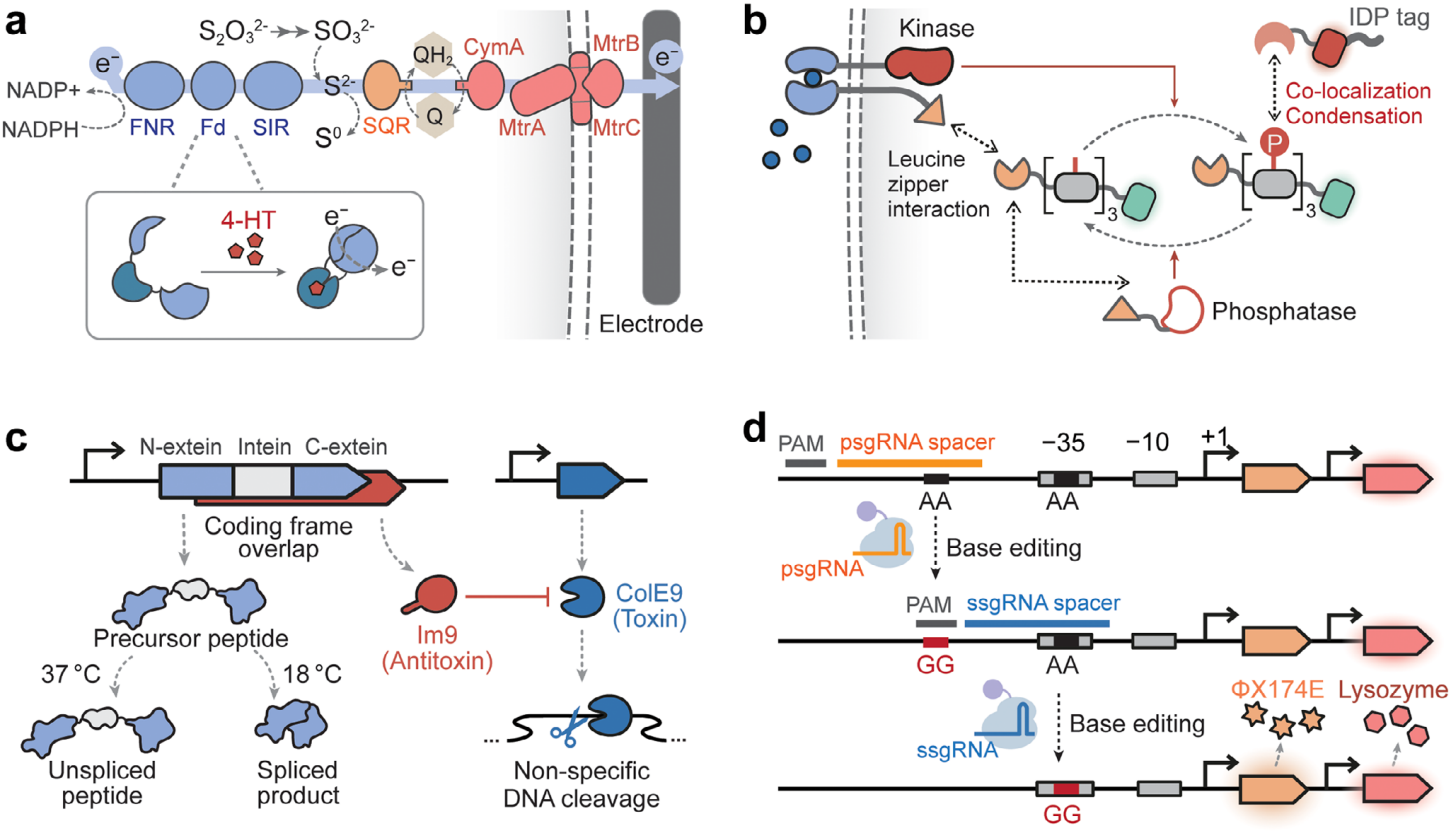
Genetic circuits for improving biosensing speed, stability, and biosafety. (a) Synthetic electron transport chain can convert the input signal (thiosulfate) into an electrical current output within two minutes. [28] The electronic biosensor can also sense 4‐hydroxytamoxifen (4‐HT) through 4‐HT‐responsive allosteric regulation of ferredoxin (Fd) conformation. [28] (b) Protein phosphorylation signaling networks for creating rapid biosensors in human cells. [196] Ligand binding to extracellular receptors activates the phosphorylation cycle, phosphorylating the substrate domain (synSub) fused to GFP. The SH2 domain fused to mCherry recognizes phosphorylated synSub, resulting in EGFP‐mCherry colocalization and intrinsically disordered protein (IDP)‐mediated condensation. (c) Gene entanglement for improving genetic stability. [29] A target gene encoding intein‐split protein is synthetically entangled with the antitoxin gene that immunizes against toxin ColE9. The split inteins sense temperature and splice to produce a functional protein at 18°C. (d) Biofuse kill switch for biosafety control. [197] Biofuse serves as a synthetic timer that activates target gene expression after a certain reaction time, defined by a series of DNA editing events. The final editing results in a modification at the –35 site of the target promoter, activating expression of the ΦX174E toxin, causing bacterial cell lysis and releasing the constitutively expressed lysozyme, which further kills neighboring cells.

Regulatory mechanisms that bypass transcription and translation have been developed for the rapid detection of target ligands. A milestone in engineering rapid biosensors is the implementation of an electron‐transfer‐based *E. coli* sensor [28], wherein a synthetic electron transport chain was constructed to convert the input signal (thiosulfate) into an electrical current output within two minutes. Furthermore, the electronic biosensor has been diversified to sense 4‐hydroxytamoxifen (4‐HT) through 4‐HT‐responsive allosteric regulation of ferredoxin (Fd) conformation (Figure 8a). Moreover, employing fluorescence aptamers as output reporters can circumvent protein translation and accelerate biosensors' response in cell‐free systems, generating immediate fluorescence upon induction, whereas fluorescent proteins require more than 9 minutes to produce clear output signals [6].

Biosensors operating at the protein level generally exhibit short response times due to the pre‐expression of protein elements. Protein phosphorylation signaling networks exhibit rapid activation/deactivation kinetics, which have been leveraged to create rapid biosensors in human cells [196]. In this system, ligand binding to extracellular receptors activates the phosphorylation cycle, which phosphorylates the substrate domain (synSub) fused to GFP (Figure 8b). The SH2 domain fused to mCherry recognizes phosphorylated synSub, resulting in EGFP‐mCherry colocalization and condensation observable within 10 minutes following the addition of the target ligand. Moreover, conditional protein cleavage was leveraged to develop the rapid protein secretion system (PASS) [198], in which input signals (e.g., chemical molecules, tumor antigens, and light) trigger dimerization of split proteases or release proteases from the cell membrane. The protease then cleaves the endoplasmic reticulum (ER) retrieval signal from the target protein, enabling its secretion within 15 minutes of receiving input signals. It is noteworthy that de novo protein design also yields a set of protein biosensors with rapid kinetics, as summarized elsewhere [199].

### 3.4 Improving Sensor Stability

The stability of biosensors is crucial for transitioning them from the laboratory to the field. Biosensors with high stability should meet two standards: (1) the biosensor should produce a robust response in the disturbances of various environmental conditions (e.g., temperature and pH); (2) the biosensors should maintain their genetic integrity for a long time, preventing the functional loss due to the accumulation of genetic mutations.

The robustness of biosensors could be improved by implementing genetic controllers [18]. Genetic controllers are genetic devices that contend with the potential effects of genetic context, cellular burden, and extracellular conditions on genetic circuit behavior, maintaining output gene expression at a relatively stable level. Genetic controllers typically adopt circuit topologies of negative feedback or incoherent feedforward loops, which can be built based on various regulatory modalities, such as antisense RNAs [200], endonucleases [201], and split inteins [202]. A representative example is the synthetic antithetic integral feedback controller based on the sigma/anti‐sigma factor interaction, which can realize perfect adaptation to intracellular disturbances (e.g., protein degradation) and environmental disturbances (e.g., temperature changes) [203].

The genetic stability of biosensors can be enhanced by designing gene entanglement and reducing metabolic burden. Gene entanglement occurs when two genes are encoded in the same DNA sequence but translated from different coding frames. Entangling a target gene with an essential gene can significantly improve genetic stability, as mutations in the target gene might affect the expression of the essential gene, leading to cell death. Leveraging this mechanism, a target gene encoding intein‐split meganuclease was synthetically entangled with the essential gene *im9* that immunizes against toxin ColE9 (Figure 8c) [29]. The temperature‐sensitive variant of L212P VMA1 intein, obtained by directed evolution, was inserted into the meganuclease to exert temperature‐dependent control over meganuclease activity [204]. The precursor peptides undergo intein‐mediated protein splicing to produce functional meganuclease for cleaving the target plasmids at 18°C, but fail to splice at 37°C. To devise synthetic gene entanglement, the *im9* gene was cloned downstream of the gene coding the thermoregulated meganuclease (TSM), utilizing a translation initiation site within the *tsm* gene. This created an N‐terminal, unstructured extension on the Im9 protein without affecting its functionality of neutralizing the toxin ColE9. The entangled meganuclease exhibited robust activity and low mutation rates, remaining genetically stable in the mouse gut over a week [29]. Notably, computational pipelines have been developed to automatically design synthetic gene entanglements [205]. Furthermore, the metabolic burden imposed by synthetic biosensors on host cells can be reduced by dividing the biosensing circuits across multiple cells [35] and by engineering synthetic differentiation [206], decoupling target gene expression from cell proliferation. More recently, novel regulatory mechanisms such as phase separation [207] and cooperative assembly [208] have emerged to enhance gene expression stability and long‐term genetic circuit stability, respectively, whose applications in biosensors are worth exploring in future studies.

### 3.5 Ensuring Sensor Biosafety

Biosafety concern is a critical bottleneck hindering the field deployment of synthetic biosensors. To address this challenge, diverse biocontainment circuits have been developed to prevent the escape of engineered cells into non‐permissive environments. For instance, a multilayered genetic safeguards system was designed by integrating riboregulated expression of essential genes, auxotrophy, and a synthetic addiction module based on genome‐cleaving endonucleases to restrict the engineered bacteria stringently in a permissive environment with an escape frequency below 1.3 × 10^−12^ (around 10^4^‐fold below the NIH standard, 10^−8^) and remained functional stable after 14‐day growth in permissive conditions [209]. Another biocontainment circuit leveraged CRISPR/Cas9‐mediated cleavage of the bacterial genome to design a kill switch responsive to both chemical signals and temperature, enabling the in situ elimination of intestinal bacteria by chemicals and the spontaneous killing of excreted bacteria via temperature changes [210]. This kill switch exhibited robust activity in the mouse gut, an escape frequency below 10^−8^, and significant long‐term stability, maintaining killing capacity after continuous growth for 28 days. The other biocontainment circuits include the Deadman and Passcode kill switches (with escape frequency below 10^−7^) [30], the pH‐sensitive kill switch (with escape frequency below 10^−11^ after 100 generations of growth) [211], and the GeneGuard systems (designed for preventing horizontal gene transfer of synthetic circuits) [212].

In addition to spatial confinement, biosafety control can also be achieved by manipulating the lifespan of the engineered cells. Recently, Huang et al. [197] developed a delayed bacterial autolysis system based on Biofuse. Serving as a synthetic timer, Biofuse can activate or repress target gene expression after a certain reaction time, comprising a series of DNA cassettes that can be sequentially edited by the adenine base editor (Figure 8d). The final editing results in a modification at the −35 site of the target promoter, activating expression of the ΦX174E toxin, causing bacterial cell lysis and releasing the constitutively expressed lysozyme, which further kills neighboring cells. Notably, the reaction time of Biofuse can be tuned from hours to days, allowing biosensors to self‐destruct after completing the detection tasks.

## 4 Empowering Synthetic Biosensors With Novel Functionality

### 4.1 Synthetic Signal Processing

Incorporation of synthetic signal processing into biosensors can reshape their dose‐response curves, accelerate sensor response, and enhance sensing specificity and stability (as discussed in Section 3). Beyond improving sensor performance, synthetic signal processing can empower biosensors with novel functionality, such as memory and logic computation [18].

#### 4.1.1 Synthetic Memory

Synthetic memory allows biosensors to record intermediate changes in cell states and transient stimuli in dynamic environments, such as the human gut. Synthetic memory can be implemented using bistable genetic switches (e.g., toggle switches [8, 48] and positive feedback loops [208, 213]) or DNA modification by recombinases [8, 214], retron [215], and CRISPR/Cas systems [216].

**FIGURE 9:**
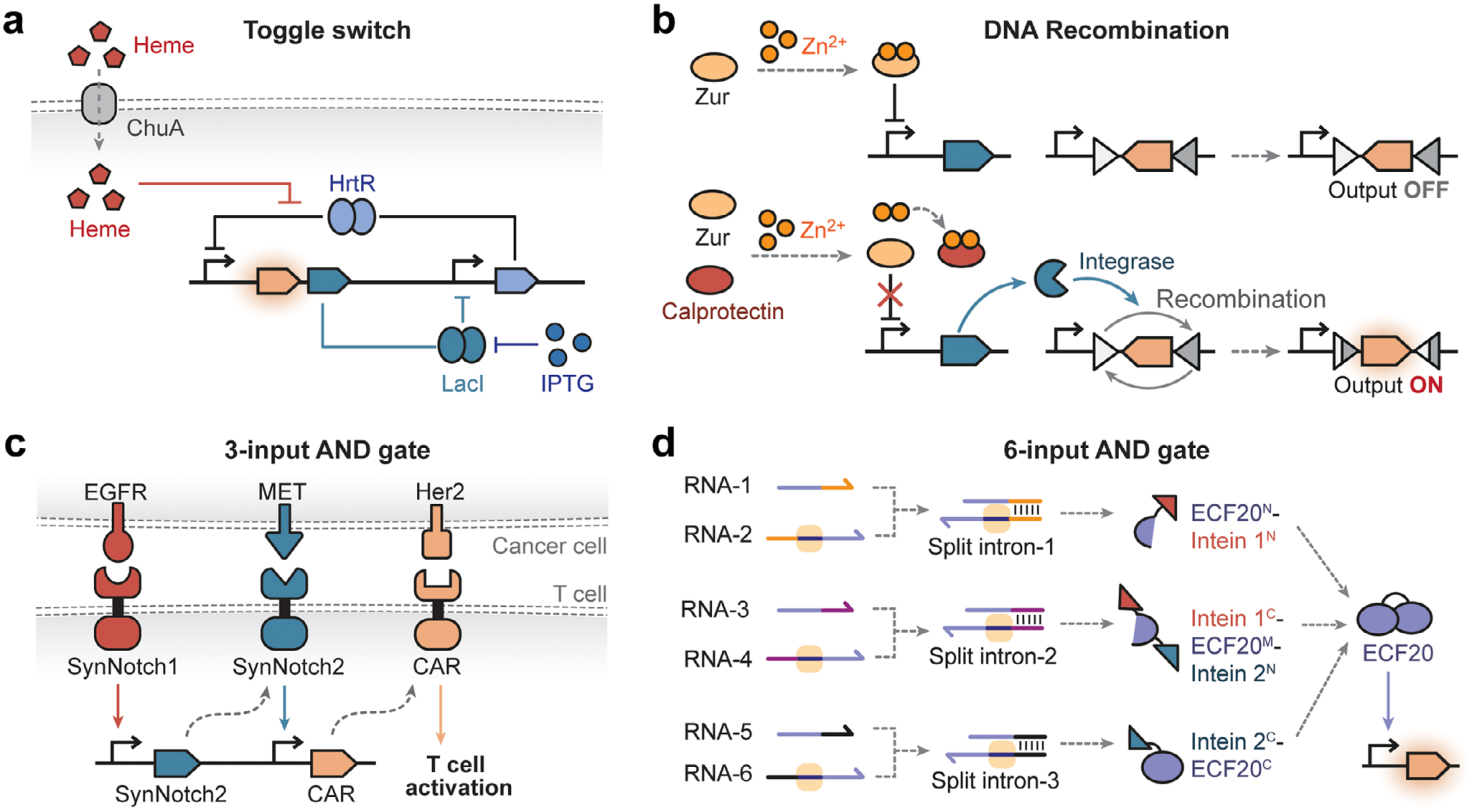
Achieving synthetic memory and logic computation in synthetic biosensors by genetic circuits. (a) Genetic toggle switch comprises two repressors, HrtR and LacI, that mutually inhibit each other for implementing synthetic memory. [48] (b) Recombinase‐based memory devices mediate DNA inversion or excision in response to input signals. The target ligand, calprotectin, competes with Zur for binding Zn^2+^, releasing the promoter from transcriptional inhibition and driving the integrase expression, leading to DNA inversion and activation of the reporter gene. [217] (c) The three‐input AND gate detects three antigens on the cancer cell membrane, activating T cells to selectively kill cancer cells. [221] (d) The split‐biomolecule‐enabled six‐input AND gate simultaneously processes six input RNA signals. [139] The input RNAs first undergo three RNA splicing reactions catalyzed by three orthogonal introns, producing three mRNAs, translated into three peptides that trans‐splice into a functional transcription activator, ECF20, activating downstream gene expression.

The most classic memory circuit is the genetic toggle switch, which comprises two regulators mutually repressing each other. The genetic toggle switch has two stable states in which one gene dominantly inhibits the other, and, once integrated into biosensors, can switch from one state to the other in response to input signals. For instance, the tetrathionate biosensor based on the TtrRS two‐component system was used to trigger the state transition of a toggle switch, retaining memory for seven days after signal removal [8]. More recently, the heme‐responsive repressor HrtR and IPTG‐controllable LacI were utilized to deploy a genetic toggle switch for early detection of fecal occult blood (Figure 9a) [48].

Transient input signals can also be recorded in DNA sequences. Recombinases can mediate DNA inversion or excision between specific recognition sites, changing the orientation or presence of regulatory elements such as promoters, RBSs, and terminators. Recently, a Zur‐responsive promoter was linked to the recombinase‐based memory circuit to detect and record intestinal inflammation in vivo [217]. In this circuit, Zur can bind with zinc to inhibit the output promoter, while the target ligand, calprotectin, competes with Zur for binding with zinc, releasing the promoter from transcriptional inhibition to drive the expression of integrase, leading to the inversion and activation of the reporter gene (Figure 9b). Likewise, a thermal‐sensitive biosensor was coupled with recombinases to enable focused ultrasound activation of long‐term therapeutic gene expression and to suppress tumor growth [214]. It has been shown that recombinase‐based memory devices can stably store information for three weeks in human urine and blood [218].

DNA cleavage and editing can also be used to record input signals, leading to two multiplexed analog cellular recoding CAMERA systems [216]. The CAMERA 1 system comprises three plasmids: the writing plasmid, which expresses the gRNA and Cas9, as well as the recorder plasmids 1 (R1) and 2 (R2). The gRNA‐Cas9 selectively cleaves R1, decreasing the R1:R2 ratio. The CAMERA 2 system employs base editing to encode the input signal into plasmids or the genome DNA in bacteria or human cells. Both CAMERA systems can sense and record the duration, magnitude, and order of diverse input signals, including antibiotics, nutrients, phage infection, light, and Wnt signaling stimulus. Transient expression of endogenous transcripts can also be captured and recorded by the reprogrammed tracrRNA‐guided base editor, leveraged to recode mobilized antibiotic resistance in *E. coli* and infection‐induced sRNAs in *Salmonella enterica* [162].

#### 4.1.2 Logic Computation

Logic computing circuits enable customization of output patterns based on arbitrary input combinations, as defined by the truth table. Typical two‐input Boolean logic gates include AND, OR, NIMPLY, NAND, NOR, IMPLY, XOR, and XNOR gates, of which the former three have been more extensively studied for integration into synthetic biosensors. These basic logic gates can be wired to form complex combinatorial circuits that process multiple input signals.

The logic computation capabilities enable biosensors to integrate multiple signals for accurate decision‐making in complex environments, enhancing disease diagnosis accuracy, improving the specificity of cell therapies, and elevating bioproduction yields. The diagnosis specificity for gut inflammation can be improved through an AND gate for simultaneous sensing of thiosulfate and nitrate [219]. The tumor‐targeting specificity of engineered bacteria can be increased by an AND‐gate architecture that expresses essential genes in response to both hypoxia and lactate [220]. Moreover, precise recognition of target cells by T cells can be achieved through a three‐input AND gate that simultaneously detects three antigens on the target cell membrane, thereby activating T cells to selectively kill target cells (Figure 9c) [221]. Similarly, logic‐gated CAR‐natural killer cells that recognize three antigens via an OR‐NOT gate can avoid on‐target/off‐tumor toxicity in vivo, enabling precise killing of tumor cells [222]. For metabolic engineering, genetic AND gate sensing oxygen and glucose can be used to dynamically regulate endogenous metabolic pathways during bioproduction [223].

Currently, complex logic circuits are designed mainly by transcriptionally concatenating simple logic gates such as NOT and NOR gates. The automatic design of such circuits can be completed using Cello [224], a software that generates tailored circuit designs from user‐defined truth tables and maps the circuits into DNA sequences across diverse host chassis [225, 226]. However, the layered design of transcriptional circuits suffers from long response times, large genetic footprints, and heavy host burdens, hindering their applications in biosensor development. To address these issues, regulatory modalities at the transcriptional, post‐transcriptional, translational, and post‐translational levels can be coupled into multi‐level hybrid circuits for complex logic computation [18] One representative example is the split‐biomolecule‐enabled six‐input AND gate [139]. In this six‐input AND gate, the input RNAs first undergo three RNA splicing reactions catalyzed by three orthogonal introns, producing three mRNAs, translated into three peptides that trans‐splice into a functional transcription activator, ECF20, activating downstream gene expression (Figure 9d). Building on well‐characterized libraries of orthogonal introns and inteins, this split‐biomolecule‐enabled logic circuit is easy to construct and genetically compact (with total circuit size around 5.5 kb), operates at a fast timescale (with 10‐fold responses after 4h induction), and increases the signal‐processing capacity of a single transcription factor by 10‐fold, establishing a novel paradigm for logic computing circuit design. Although not evaluated in mammalian systems yet, this design holds great promise for cross‐kingdom regulation, as both split introns and inteins have been reported to be portable across diverse host organisms, including bacteria, yeast, plants, and mammalian cells [227, 228, 229].

### 4.2 Multimodal Biosensor Output

Most synthetic biosensors rely on fluorescent reporters, which require specialized equipment for fluorescence measurement, limiting their field applications. To overcome this bottleneck, novel output modules and portable instruments have been devised for decentralized applications of synthetic biosensors. The advancements include the visualization of biosensor outputs as easy‐to‐interpret spatial patterns and the conversion of output signals into forms detectable by portable devices.

#### 4.2.1 Output Visualization and Spatial Patterns

Synthetic biosensors can be programmed to display spatial patterns visible to the naked eye or on cell phones. A microbial sensor cell array, for instance, can be created by using agarose hydrogel entrapment or microfluidic encapsulation to align multiple biosensors with varying detection limits [5]. Upon the addition of target ligands, this sensor array displays an intuitive volume bar‐like pattern, which can be captured by a cell phone, to indicate the pollutant level in groundwater samples. Similar patterns can also be generated via a strip of tests of cell‐free biosensors with varying detection thresholds [169]. Moreover, colorimetric output modules (e.g., β‐galactosidase [7], flavin monooxygenase [138], violacein biosynthesis pathway [230]) reflect the target concentrations via color changes, thereby visualizing the detection results of synthetic biosensors, especially the paper‐based biosensors [45, 151]. In mammalian cells, secreted embryonic alkaline phosphatase (SEAP) offers a rapid, non‐invasive, and cost‐effective readout modality without requiring cell lysis, capable of generating colorimetric or chemiluminescent outputs, and allowing for long‐term monitoring of biosensor responses [231]. Furthermore, diverse colony patterns on solid agar surfaces can be generated by controlling bacterial swarming motility, encoding the input signals into spatial patterns [21]. The input signal information can be accurately decoded from specific spatial pattern features, such as the ring width and radial asymmetry, assisted by deep learning models.

#### 4.2.2 Interfacing Output Modules With Portable Devices

The field deployment of synthetic biosensors can also be facilitated by converting the biochemical output signals to those that can be easily detected by portable devices. A landmark is the construction of a modular, eight‐component synthetic electron transfer chain in *E. coli* that rapidly generates an electrical current upon exposure to environmental contaminants (e.g., thiosulfate and 4‐HT), enabling real‐time monitoring of target molecules with miniature electrodes [28].

Biosensors can be connected with electronics through biochemical transducers. For instance, the gene activation in cell‐free biosensors can be converted into a glucose signal that can be read by off‐the‐shelf glucose meters. This glucose‐meter interface has been shown to detect both chemical and nucleic acid inputs, enabling sample‐to‐result diagnosis of typhoid and paratyphoid [232]. Likewise, the phenazine biosynthesis pathway can be employed as an output module to produce phenazine‐1‐carboxylic acid (PCA), a redox‐active molecule that generates output current detectable by electrodes [233]. Moreover, the charged ions released by bacterial metabolic processes can reduce electrical current impedance [234]. Therefore, biosensor‐controlled bacterial lysis was engineered to increase impedance, which was monitored by a miniaturized electrode array.

Optical signals from fluorescent or bioluminescent reporters in biosensors can also be detected by electronic devices. A bacteria‐electronic capsule was developed to detect heme‐induced bioluminescence signals in the gastrointestinal tract and wirelessly transmit them to an external device for rapid, accurate diagnosis of porcine gastric bleeding [235]. In a following study, the capsule size was reduced to less than 1.4 cm^3^, for miniaturized wireless sensing of labile inflammatory biomarkers in live pigs [49]. Finally, a bidirectional communication between electronics and microbes is implemented, using remotely controlled LEDs in the capsule to activate bacterial expression of therapeutic genes to alleviate colitis [236].

Although a variety of reporter modules have been developed, large‐scale studies systematically evaluating these biosensors outside laboratory settings remain limited. Most biosensors were characterized by skilled experts in the laboratory using pre‐treated samples. Only recently, Thavarajah et al. reported the first field trial of biosensors operated by non‐expert users for detecting fluoride contamination of drinking water in rural Kenya [237]. This cell‐free biosensor relies on a fluoride riboswitch to regulate the transcription of a colorimetric reporter catechol (2,3)‐dioxygenase (C23DO) [128]. The freeze‐dried cell‐free reactions were rehydrated by 52 local participants, and 57 water samples from 36 households were analyzed; biosensors correctly classified elevated fluoride (above the WHO upper limit, 1.5 ppm) in 89.5% water samples [237]. For disease diagnosis, a double‐blinded field test was also reported to evaluate the paper‐based cell‐free sensors for Zika and chikungunya viruses, which leverages toehold switches to sense viral RNAs and produce colorimetric reporter LacZ [238]. The paper‐based Zika and chikungunya virus diagnostics provide specificity and sensitivity equivalent to RT‐qPCR tests, with 98.5% diagnostic accuracy across 268 patient samples. In addition to chromogenic reporters, biosensors with alternative outputs (e.g., bioluminescence [49], electrical currents [28], and hyperspectral reporters [239]) have been demonstrated for field detection or in animal models, although on a much smaller scale. Given the wealth of biosensors developed, future studies systematically assessing their performance profiles (i.e., detection accuracy, specificity, usability, cost, speed, and robustness) in field settings will facilitate their broad implementation for on‐site, democratized biosensing to address real‐world challenges.

## 5 Conclusions and Perspectives

Synthetic biology has empowered unprecedented control over biological systems and provided a rich palette of sensor, signal processor, and actuator modules for customizing synthetic biosensors. Incorporation of genetic circuits into synthetic biosensors not only provides powerful tools to elevate biosensing performance in terms of sensitivity, specificity, speed, stability, and biosafety but also extends the functionality of biosensors, enabling the recording of the transient signals and assimilating multiple signals for accurate decision making.

Synthetic biosensor development will be accelerated by the discovery of novel sensory mechanisms and the implementation of genetic circuits with unique functions. Developing novel sensory and regulatory modalities will expand the sensing scope of synthetic biosensors. For instance, the discovery of the reprogrammability of CRISPR tracrRNA‐crRNA pairs led to a series of biosensors for detecting endogenous RNA expression and viral RNA in vitro [16, 161, 162]; the de novo design of toehold switches has contributed to the emergence of paper‐based cell‐free biosensors for nucleic acid detection [7, 23, 136, 167]. Future studies exploring RNA‐ and protein‐level regulation, such as RNA‐ and protein‐splicing [45, 139], will yield synthetic biosensors with higher programmability and faster response, which will also be better suited for specific applications such as RNA therapeutics [240]. Next, incorporating genetic circuits into synthetic biosensors will further enrich their functionality. Although a wealth of genetic circuits has been utilized to enhance the sensing performance, as discussed throughout the review, many have not been tested. Bandpass and bandstop filters [172], for example, have been rarely employed in biosensors, whose signal filtering function might be advantageous for monitoring physiological biomarkers (e.g., lactate [45]), as low and high concentrations of these biomarkers both indicate health disorders.

Notably, tuning the expression levels of biosensor components and engineering biorecognition elements are still straightforward and effective ways to alter the sensitivity and specificity of biosensors. Therefore, it is necessary to establish standardized libraries and databases for modular genetic parts, including regulatory parts (e.g., promoters, RBSs, and terminators) and biorecognition elements (e.g., allosteric transcription factors and riboswitches) with high‐quality comparable performance metrics characterized in the same contexts [1, 241] Moreover, developing novel techniques for high‐throughput screening or predictably designing biorecognition elements is in great demand. For instance, identifying a new RNA aptamer that binds target ligands in vitro can be time‐consuming and laborious, and the resulting aptamer might not even be functional after integration into riboswitches. This process can be accelerated by high‐throughput platforms such as UltraSelex, a deep‐sequencing‐based single‐step method for discovering RNA aptamers in one day [242], and automated computational design tools for synthetic riboswitches [146, 147].

The field deployment and real‐world application of synthetic biosensors will benefit from future research in several directions. Comprehensive profiling of the sensitivity, specificity, speed, stability, and biosafety of synthetic biosensors is needed to systematically evaluate and optimize biosensing performance. While a wealth of genetic circuits has been constructed to improve each feature, they can hardly be integrated into a single cell while retaining their original functions due to the large circuit size and limited host resources. Therefore, it is important to explore elegant genetic circuit design that yields high‐performance biosensors while imposing a lower metabolic burden on host cells. Alternatively, interfacing biosensors with nanomaterials and electrical devices can physically encapsulate the engineered microbes for biocontainment and also enhance sensitivity, specificity, and stability by reducing environmental disturbances [28, 49, 235, 236]. Furthermore, on‐site precise conversion of biosensor output to the input concentration is needed for accurate quantification of target ligands in complex settings, which can be assisted by machine learning algorithms. For instance, the least square support vector machine (LS‐SVM) algorithm was applied to develop a discrimination model for fungal contamination in wheat. Trained on data from 70 samples (72 data points per sample), the LS‐SVM algorithm can decode detection results from a whole‐cell sensor array with 97.24% accuracy [243]. In addition, a long short‐term memory (LSTM) network was trained on the time‐dependent dose‐response curves of a gold ion biosensor and utilized to rapidly predict target concentrations at the early detection stage, achieving high precision (78%) within 30 min post‐induction [34]. These cases demonstrate the pivotal role of artificial intelligence in improving the interpretation of output signals and the efficiency of data analysis, which can be applied to numerous biosensors with open‐source code and reproducibility guidelines.

Taken together, synthetic biosensor design and field deployment will be expedited via merging the advancements in synthetic biology, materials science, electronic engineering, and artificial intelligence. The successful outcome will contribute to next‐generation biosensing systems with excellent sensitivity, specificity, speed, stability, and biosafety, revolutionizing disease diagnosis and treatment, biomanufacturing, environmental monitoring, food safety control, and diverse other fields.

## Funding

National Key R&D Program of China (2025YFA0921900) “Pioneer” and “Leading Goose” R&D Program of Zhejiang (2024C03011, 2025C01097) National Natural Science Foundation of China (32271475, 32320103001, 32501287)

## Conflicts of Interest

The authors declare no conflicts of interest.

## Data Availability

The authors have nothing to report.
